# Outcomes and outcome measurement instruments in lower-limb lengthening surgery: a scoping review to inform core outcome set development

**DOI:** 10.2340/17453674.2024.42488

**Published:** 2024-11-29

**Authors:** Ali YALCINKAYA, Ole RAHBEK, Maria TIRTA, Jette Frost JEPSEN, Michael Skovdal RATHLEFF, Christopher IOBST, Søren KOLD

**Affiliations:** 1 Interdisciplinary Orthopaedics, Aalborg University Hospital, Aalborg, Denmark; 2 Department of Orthopaedic Surgery, Aalborg University Hospital, Aalborg, Denmark; 3 Medical Library, Aalborg University Hospital, Aalborg, Denmark; 4 Department of Physical Therapy and Occupational Therapy, Aalborg University Hospital, Aalborg, Denmark; 5 Department of Health Science and Technology, Faculty of Medicine, Aalborg University, Aalborg, Denmark; 6 Center for Limb Lengthening and Reconstruction; Nationwide Children’s Hospital, Columbus, OH, USA

## Abstract

**Background and purpose:**

The heterogeneous outcomes used in lower-limb lengthening surgery (LLLS) complicate evidence synthesis, weakening systematic reviews and clinical recommendations, and reducing research impact. This scoping review maps the outcomes and outcome measurement instruments (OMIs) used in LLLS.

**Methods:**

This pre-registered review systematically identified studies reporting outcomes in adults or children who underwent LLLS. Outcomes and OMIs were extracted verbatim, and experts grouped outcome terms under headings using the COMET taxonomy.

**Results:**

The search found 5,308 unique hits, including 149 studies from 2020–2024. They reported 2,939 verbatim outcomes, which were consolidated into 92 outcome headings and 27 subheadings. “Life impact” accounted for 13%, while “Clinical outcomes” represented 83% of all outcomes. Among the clinical outcomes, “Musculoskeletal and connective tissue” was the most reported outcome domain (68% of all outcomes). The most frequently reported outcomes were “Lengthening,” “Bone healing,” “Range of motion,” “Limb alignment”, and “Complications.”

**Conclusion:**

Outcomes reported for people undergoing LLLS are heterogeneous and vary widely in the definitions and measurement tools used to collect them. Outcomes likely to be important to patients (life impact outcomes), such as quality of life and measures of physical function, are rarely reported.

Lower-limb lengthening surgery (LLLS) has continued to evolve from the introduction of distraction osteogenesis by external fine-wire circular fixation to the development of hexapod computer-assisted circular fixator to the current all internal motorized intramedullary lengthening nails [1]. Despite a lack of prospective comparative studies, an increasing proportion of patients are treated with intramedullary lengthening nails instead of external fixators [2]. However, whether intramedullary nails are beneficial compared with external fixators might depend on which outcome is studied, as both improving [3] and negative [4] effects have been shown.

A core outcome set (COS) is a minimal set of outcomes to be measured and reported in all clinical studies for a specific health condition [5]. These sets standardize outcome measurement across studies, facilitating comparisons and meta-analyses, thereby improving clinical guidance [6]. The lack of a universally accepted COS in LLLS causes variability in outcomes across studies, and limits evidence synthesis when performing systematic evaluations of surgical effectiveness and safety [7-11]. Clinician and outcome reporting biases also pose significant problems by considerably affecting the validity and generalizability of study findings [12-14].

To tackle these issues, a scoping review (a type of evidence synthesis aimed at mapping the breadth and depth of research on a specific topic) was used to explore outcomes and outcome measurement instruments (OMIs) in LLLS literature. The findings may influence future COS development for LLLS. The establishment of a standardized outcome set has the potential to improve reporting consistency and boost evidence quality [13].

## Methods

All processes were conducted and reported in accordance with the JBI methodology and the PRISMA-ScR guidelines [15,16].

### Eligibility criteria

All peer-reviewed studies focused on any aspect of LLLS were included. Studies were excluded if LLLS on human subjects was not the primary focus or relevant data could not be extracted. Editorials, conference abstracts, and non-English articles were also excluded.

### Information sources and strategy

We included all studies published until May 22, 2024. Only articles in English were searched and considered for inclusion. The search of peer-reviewed papers in MEDLINE, Embase, Cochrane Library, Web of Science, and Scopus databases were performed in a systematic fashion described below.

Due to the high number of articles identified in the search and considering the improvements in surgical methods over time, it was deemed appropriate to deviate from the protocol and use the “data saturation” strategy to control article inclusion. This method involved extracting outcomes on a year-by-year basis, starting from the most recent year and going back until no new outcome headings could be extracted from an entire year of studies. This strategy yielded outcomes from studies published from 2020 onward.

A confirmatory analysis was conducted to ensure no outcomes were missed. We reviewed outcomes from the most frequently cited articles from each 5-year period between 1995 and 2020. This confirmed that data saturation was achieved, capturing all relevant outcomes without missing any important data.

### Search strategy

Relevant keywords and MeSH terms were used from inception to May 2024, limited to peer-reviewed publications for high-quality data (Appendix 1).

### Selection of sources of evidence

Article screening was performed using Rayyan software (rayyan.ai) [17]. 2 reviewers (AY, MT) independently screened articles, with full texts of potentially relevant ones considered for inclusion. Disagreements were resolved by consensus or a third reviewer (SK, OR, CI).

### Data items and charting process

2 reviewers (AY, MT) independently charted the data using an Excel spreadsheet (Microsoft Corp, Redmond, WA, USA) and a REDCap form [18,19] (Appendices 2.1 and 2.2). Disagreements were resolved by consensus or a third reviewer (SK, OR, CI).

All outcomes were extracted verbatim alongside any associated OMIs used. All outcomes and OMIs were extracted from the abstract, methods, or results section of each included study. Outcomes were defined if an outcome definition was provided or referenced in a citation. If an outcome definition was directly linked to a citation, the outcome definition was extracted verbatim from the source or original publication. The data fields extracted from the full-text review are indicated in Table 1, and the extraction steps are explained in Table 2.

**Table 1:** Data fields extracted at full-text review

Study demographics
Publication date
Study title
Intervention type
Intervention site
Journal
Location (country where research was conducted)
Study design (e.g., RCT, cohort, case series)
Study population
Number of participants
Sex distribution of participants
Age distribution of participants
Study inclusion and exclusion criteria
Follow-up duration
Study outcomes
Primary/secondary outcomes identified/defined
Outcome wording (extracted verbatim)
Outcome definition
Outcome measurement instruments used
Subdomains covered by outcome

RCT = randomized controlled trial.

**Table 2:** Outcome extraction methodology

Order	Extraction steps	Description
1	Verbatim extraction of outcomes	
	Word-for-word extraction directly from the included studies	
2	Mapping the outcomes under newly created outcome headings	
	New, overarching outcome headings created to bundle outcomes of the same/similar scope	
3	Outcome headings categorized using the 38-scale COMET taxonomy of outcomes	
	Outcomes placed under the relevant outcome domains and core areas according to the framework	

COMET = Core Outcome Measures in Effectiveness Trials.

Verbatim names of the patient-reported outcome measures (PROMs) and all their components including individual items, additional non-validated questions, and developers’ scale definitions were extracted and categorized as described in Table 3.

**Table 3:** PROMs extraction methodology

Order	Description of extraction steps
1	Identification of PROMs used in the included studies
2	Detailed content analysis to examine the scale components and all single items included in each PROM
3	Categorization of PROM content into health domains according to the COMET taxonomy of outcomes

COMET = Core Outcome Measures in Effectiveness Trials;

PROM = patient-reported outcome measure.

### Synthesis of results

Our outcome categorization primarily follows the COMET framework, where extracted outcomes, OMIs, and PROMs were analyzed and mapped into broader outcome domains and core areas [20]. Additionally, we integrated the OMERACT Filter 2.0 to enhance consistency and comprehensiveness [21]. For the Life impact area, we used WHO-ICF classifications, aligning with OMERACT’s guidance [21-24]. Initial mapping was done by AY, with consensus reached by involving SK, OR, MSR, and CI.

For visualization, the web application Flourish (available from https://app.flourish.studio/login) was used [25].

### Terminology

This review’s hierarchical classification includes Core areas, Outcome domains, Outcome headings, Subheadings, and Verbatim outcomes, based on the COMET taxonomy with modifications from Aquilina et al. [26,27].

- Core areas: Frameworks organizing outcome domains into 5 categories—death, adverse events, life impact, physiological/clinical, and resource use—that cover all critical aspects of a health condition or intervention [6,20,21].
- Outcome domains: Specific aspects within core areas, categorized into 38 types according to the COMET taxonomy [6,20].
- Outcome headings: Broad subcategories within outcome domains, refined for precision. Similar outcomes are merged under single headings, with some including subheadings for added detail [20,26,27].
- Verbatim outcome: In clinical trials, an outcome measures treatment effects, including side effects (risk) or effectiveness (benefit) [6]. Outcomes extracted directly from articles are called verbatim outcomes.
- Outcome measurement instruments (OMIs): Tools or definitions used to evaluate the quality or quantity of outcomes [26].

### Registration, use of AI, funding, and disclosures

The scoping review was registered with the Open Science Framework, and its protocol is available on the platform [28]. The authors received no financial support for the research, authorship, and/ or publication of this article. There was no use of artificial intelligence (AI) for the production of the current study. The authors declare no conflicts of interest. Complete disclosure of interest forms according to ICMJE are available on the article page, doi: 10.2340/17453674.2024.42488

## Results

### Study selection

We identified 12,402 articles initially. After removing duplicates, 5,308 articles were screened, resulting in 1,054 for full-text review with a conflict rate under 5%. Data extraction reached saturation in 2020, leaving 149 articles for inclusion in the review (Figure) [29].

**Figure:**
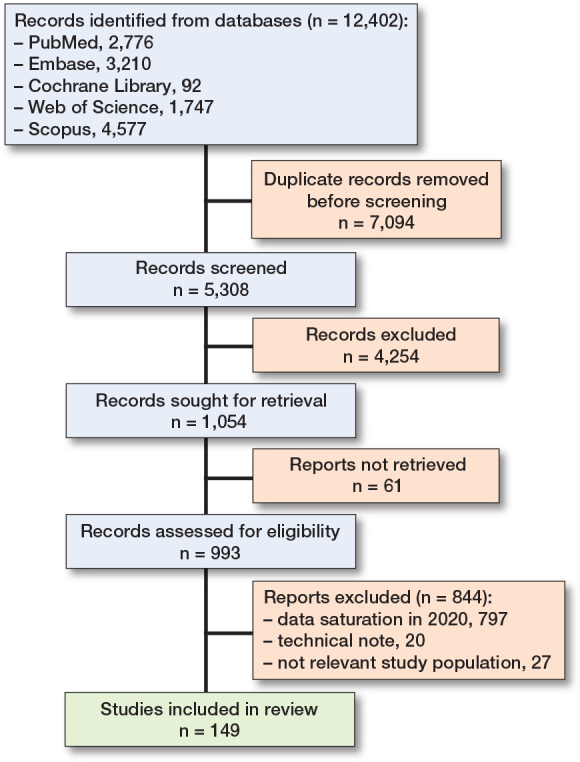
Flowchart of study selection.

### Characteristics and scientometrics of included studies

149 studies were included in this scoping review, with publication years ranging from 2020 to May 22, 2024. The number of studies published each year was relatively evenly distributed across this period but with diverse study designs (Table 4).

**Table 4:** Summary characteristics and scientometrics of included studies (N = 149). Values are count (%)

Publication year	
2020	30 (20)
2021	33 (22)
2022	39 (26)
2023	30 (20)
2024 (to May 22)	17 (11)
Study design	
Case report	16 (11)
Case series/ database reviews	98 (66)
Retrospective cohort study	21 (14)
Prospective cohort study	6 (4)
Randomized controlled trial	1 (1)
Case-control	3 (2)
Cross-sectional	4 (3)
Top 6 journals of publication	
Journal of Pediatric Orthopaedics	13 (9)
Strategies in Trauma and Limb Reconstruction	11 (7)
Journal of Limb Lengthening & Reconstruction	9 (6)
Acta Orthopaedica	7 (5)
Children (MDPI)	6 (4)
Orthopaedics & Traumatology: Surgery & Research	6 (4)
Top 6 countries of origin	
USA	44 (30)
Germany	8 (5)
China	8 (5)
Turkey	8 (5)
UK	7 (5)
Russia	7 (5)
Study centers	
Single center	131 (88)
Multicenter	18 (12)

Most studies were single center (131 studies, 88%), primarily consisting of case series or database reviews (98 studies, 66%), followed by retrospective cohort studies (21 studies, 14%), and case reports (16 studies, 11%).

The geographic origin of the studies included multiple countries, with the USA contributing the highest number (44 studies, 30%).

### Orthopedic device(s)

Various implants and techniques were noted (Table 5), with motorized/magnetic lengthening nails being the most common (48%, n = 71). Traditional circular fixators were used in 30% (n = 44), while monolateral fixators and hexapod fixators appeared in 20% (n = 30) and 19% (n = 28) of the studies, respectively.

**Table 5:** Orthopedic device(s) type reported in included studies (N = 149)

Device type	n (%)
Motorized/magnetic lengthening nails	71 (48)
Traditional circular fixators	44 (30)
Monolateral fixators	30 (20)
Hexapod fixators	28 (19)
Lengthening then nailing	9 (6.0)
Non-motorized lengthening nails	3 (2.0)
Other	18 (12)

### Outcome categorization and frequency of reporting

We extracted 2,939 individual outcomes verbatim from the literature on LLLS. These outcomes were grouped into 92 outcome headings (“Outcome domains” in OMERACT terms), representing similar meanings or scopes of measurement, and further divided into 27 subheadings. They were analyzed and categorized according to the COMET taxonomy [20], resulting in classification under 19 overarching outcome domains and 4 core areas: “Adverse events,” “Life impact,” “Physiological or clinical,” and “Resource use.” The core area “Death” was excluded, as LLLS rarely impacts lifespan. Adverse event outcomes that were specifically named were appropriately categorized under the relevant category and identified as harm outcomes [30]. A detailed breakdown of these outcomes, domains, specific headings, and subheadings is provided in Appendix 3 (Interactive map: https://public.flourish.studio/visualisation/18576652/).

Life impact outcomes (“Life impact” in OMERACT terms, or “Activities and participation” in the ICF model) comprised 13% (384 out of 2,939) of the total outcomes. The physical functioning outcome domain comprised 16 outcome headings and 15 subheadings used to evaluate patients’ physical function.

Physiological or clinical outcomes (“Pathophysiological manifestations” in OMERACT terms) alone accounted for 77% (2,252 out of 2,939) of the total outcomes while reporting of adverse events accounted for 7% (194 out of 2,939). Combined, these categories account for 83% (2,446 out of 2,939) of the total outcomes.

Classified under the “Physiological or clinical” core area, musculoskeletal and connective tissue outcomes accounted for 68% of all outcomes. The most frequently reported outcomes included “Lengthening,” “Bone healing,” “Range of motion,” and “Lower limb alignment.” Additionally, complications were reported in 138 occurrences (Table 6).

**Table 6:** The 10 most common outcomes reported in included studies (N = 149)

Core area Outcome domains Outcome headings	Number of verbatim outcomes
Adverse events	
Adverse events	
Complications	138
Physiological or clinical	
Musculoskeletal and connective tissue outcomes	
Lengthening	456
Bone healing	236
Range of motion	215
Lower limb alignment	196
Nonunion or nonhealing (harm)	151
Hardware or implant failure (harm)	133
Pain or discomfort	110
Time in/with hardware	96
Deformity	76

### Outcome measurement instruments

We identified and cataloged 199 distinct OMIs. These included 133 specific outcome definitions, 36 patient-reported outcomes, and 30 clinician-reported outcomes. Detailed characteristics of these instruments, along with the frequency of reporting and source text citations, can be found in Appendix 4. Of the 92 outcome headings, 33 (35%) were defined. Most definitions, totaling 139 (70%), related to the physiological or clinical core area and focused solely on objective clinical outcomes. Each definition was uniquely worded, even when describing the same outcomes. “Bone healing” was the most frequently defined outcome, appearing in 108 studies (72%) with 49 distinct measurement instruments. Either a definition, an OMI, or both were specified in 61 studies. There was considerable variation in definitions, with “Bone healing” being described in several ways. The most common definition was: “When 3 of the 4 cortices of the bone at the lengthening site were bridged with a solid white continuous cortical line, the regenerate was declared united.” Additionally, “Bone healing” was used synonymously with terms such as “Union,” “Complete consolidation,” “Osseous consolidation,” “Regenerate healing,” “Corticalization,” “Maturation of bone regenerate,” “Mature bridging callus,” and “Continuous column of bone.”

A considerable overlap in definition was noted for several outcomes, including “Bone healing,” “Time in/with hardware,” and “Full weight bearing.” The earlier definitions of bone healing and its variations were frequently used interchangeably to denote “time of external fixator removal” and “full weight-bearing time,” without clearly distinguishing whether these referred to external fixator-based or intramedullary nail-based techniques. In clinical practice, these terms do not always align with the same time points in the treatment process.

66 distinct instruments for measuring patient- or physician-reported outcomes were utilized across the included studies, primarily focusing on objective clinical outcomes. Some instruments also measured life impact, particularly in terms of physical functioning and overall health-related quality of life (HRQoL). The most used instrument in this domain was the Association for the Study and Application of the Methods of Ilizarov (ASAMI) functional criteria [31] , which was reported 9 times. HRQoL was evaluated in 12 studies (8%) using 11 different instruments (6% of the identified instruments). The most frequently used HRQoL instruments were that of the Limb Deformity-Scoliosis Research Society (LD-SRS) [32] , used 3 times for adults, and the Pediatric Quality of Life Inventory version 4.0 generic core scales (PedsQL) [33], used twice for the pediatric group.

Of note, we also found a study evaluated children’s preferences and satisfaction with lower extremity lengthening using a non-validated tool called “The Patient Questionnaire for Pediatric and Adolescent Limb Lengthening Surgical Technique and Treatment Course” [34].

### Complication classification systems

Our scoping review identified various classification systems for LLLS complications, with the Paley classification [35] most cited in 28 studies and the Lascombes classification [36] in 9 studies. A detailed breakdown of the complication classification systems is provided in Appendix 4.

### Findings on outcome specification and study criteria

We found significant variability in the reporting of outcome specifications and study criteria. Only 27 studies (18%) specified primary outcomes, and 8 studies (5%) detailed secondary outcomes, leaving 114 studies (77%) without clear definitions. Additionally, 31 studies (21%) lacked clear inclusion or exclusion criteria.

## Discussion

This scoping review is the first to map outcomes reported in LLLS systematically. We identified 2,939 verbatim outcomes, mapped into 92 headings and 27 subheadings across 19 domains and 4 core areas. Our findings reveal significant variability in the outcomes reported across all domains, with many different outcome terms grouped under the same heading. Additionally, various definitions and measurement instruments, most of which were used only once, were employed for the same outcomes. This variability complicates the comparison of results across studies and is sometimes further hindered by the use of non-validated instruments.

Nearly half of all identified outcomes were objective clinical measures, such as bone lengthening, healing, nonunion, range of motion, lower limb alignment, and infection outcomes. This highlights a clear preference for outcomes that are especially relevant to surgeons, reflecting an emphasis on outcomes that are easily quantifiable in routine practice. Despite guidance and research trends advocating for the inclusion of PROMs, the clinician-based objective measures continue to dominate [37-39]. Patient-reported outcomes (PROs) are crucial for capturing patients’ subjective experiences during LLLS. The importance of incorporating patient-centered outcomes in orthopedic surgery was stated in 1999, emphasizing that clinical outcomes should be complemented by assessments of functional outcomes, including physical function, pain, health status, work activity, and daily living activities [37]. Despite these recommendations made over 25 years ago, the limited use of patient-centered outcomes persists [38-40]. Our review further highlights this situation, showing that life-impact outcomes represented only 13% of reported outcomes, while clinical outcomes accounted for 83%. These findings highlight a strong emphasis on physiological or clinical outcomes and adverse events in the literature, potentially overshadowing the substantial life impact these surgeries have on patients. This underrepresentation of life-impact outcomes is significant, given the substantial impact this type of surgery has on patients’ lives. Although they are relatively rare, the numerous outcome headings and subheadings attempting to represent the physical functioning domain (16 outcome headings and 15 subheadings) create significant challenges in synthesizing evidence and making reasonable comparisons from the pooled data.

More than half of the studies in this review utilized circular fixators, which are often used for several months [38]. This prolonged use can significantly impact both short- and long-term health and quality of life (QoL), as fixators uniquely interfere with daily functioning [41]. Additionally, research on pediatric patients indicates a risk of negative psychological impacts, emphasizing the importance of mental and physical support from parents, family, and healthcare professionals [42]. These findings suggest that outcomes in this patient group encompass a wide range of factors that cannot be fully captured by clinical measures alone, indicating the need for patient-centered outcomes [38]. However, our review show that these outcomes are rarely reported.

A significant variation was observed in the outcome measurement tools used, encompassing both patient-reported and physician-reported metrics. Most tools focused on objective clinical measures, with limited attention to patient impact. For instance, QoL was assessed in just 12 studies, and the instruments used varied widely. 11 different HR-QoL measures were identified, highlighting this inconsistency. Despite its rare assessment, the variety of reasons for LLLS is an important factor demonstrating the need for a standardized assessment of QoL to evaluate surgical effects across different cases [39].

Adverse events, specifically reported with the wording complication, comprised 7% of reported outcomes in LLLS. However, the lack of uniformity in complication classification negatively impacts the assessment of surgical outcomes and limits evidence synthesis [8]. Our review shows that of the 149 studies, only 55 explicitly mentioned the classification system used for complications, with half of these employing the Paley classification system, while the remainder used various other systems. Although the Paley classification system appears to have gained relatively widespread adoption in this context, the criticality of complications necessitates the creation of standardized assessment and reporting methods in order to facilitate effective comparisons of complications across LLLS studies.

### Limitations

In line with COMET guidance to maintain focus and manageability [6], we limited our inclusion to studies published after 2020, employing a data-saturation strategy. This timeframe captures advancements in emerging technologies, such as intramedullary lengthening nails and computerized external fixators. While these technologies are tangential to the measured outcomes and have no direct relationship to the techniques used, their inclusion aligns with the forward-looking nature of the COS initiative. Excluding pre-2020 studies may theoretically omit rare outcomes; however, this is unlikely, given the cumulative nature of scientific research and the reliance of recent studies on prior findings. Focusing on recent years is also expected to result in more articles incorporating patient-reported outcomes (PROs), which reflects contemporary research trends. A confirmatory analysis of highly cited articles from 1995 to 2020 further ensured that no relevant outcomes were missed.

Despite capturing all relevant outcomes, our review may not fully reflect the lived experiences of patients undergoing LLLS, an area to be explored in an upcoming qualitative study [24]. Furthermore, restricting the review to English-language studies may limit its generalizability. The included studies predominantly represent high-income countries, particularly the USA (44 studies, 29%), with limited representation from lower/middle-income nations, highlighting the resource-intensive requirements of LLLS [43,44].

Although subjective mapping of outcomes using the COMET taxonomy may introduce minor inconsistencies, this framework effectively identifies knowledge gaps. Any gaps would have indicated incomplete data saturation. However, an analysis of outcome distribution within the COMET domains confirmed that all anticipated outcomes were present and appropriately mapped, demonstrating the comprehensiveness of this study. Finally, the reliance on retrospective data, with 88% of studies being single-center and including only 1 RCT, underscores a lack of high-level evidence.

### Conclusion

We showed deficiencies in outcome reporting for LLLS, especially in terms of outcome variability and the absence of patient-centered measures. It is crucial to reach a consensus among researchers and clinicians on defining and measuring outcomes. There should be a greater emphasis on quality-of-life outcomes, alongside exploring qualitative patient experiences and actively involving patients in developing a COS. Establishing and implementing a wide consensus for this COS to standardize outcome measurement might enhance evidence quality and improve management practices. The outcomes identified in this scoping review will be incorporated into a series of Delphi consensus processes to achieve this goal.

### Supplementary data

Appendices 1–4 are available on the article homepage, doi: 10.2340/17453674.2024.42488

## Supplementary Material











## Notes

AY (Primary investigator): conceptualization, screening of studies, data extraction, data mapping/analysis, drafting the manuscript. MT: screening of studies, data extraction. JPJ: building the search string, performing the literature search. CI, MSR, OR, and SK: conceptualization, screening of studies, critical revision of manuscript. All authors have contributed to this study, revised, and accepted the manuscript as submitted.

The authors sincerely thank Arash Ghaffari, Hans-Christen Husum, and Ahmet Yalcinkaya for their valuable contributions to this article. Their support and insights were greatly appreciated.

Handling co-editors: Ilkka Helenius and Robin Christensen

*Acta* thanks Franck Accadbled and John Gerard Birch for help with peer review of this manuscript.

## References

[1] Birch J G. A brief history of limb lengthening. J Pediatr Orthop 2017; 37 Suppl 2(6): S1-8. doi: 10.1097/BPO.0000000000001021. 28799987

[2] Mittal A, Allahabadi S, Jayaram R, Nalluri A, Callahan M, Sabharwal S. Trends and practices in limb lengthening: an 11-year US database study. Strategies Trauma Limb Reconstr 2023; 18(1): 21-31. doi: 10.5005/jp-journals-10080-1574. 38033925 PMC10682549

[3] Horn J, Grimsrud Ø, Dagsgard A H, Huhnstock S, Steen H. Femoral lengthening with a motorized intramedullary nail. Acta Orthop 2015; 86(2): 248-56. doi: 10.3109/17453674.2014.960647. 25191936 PMC4404780

[4] Hlukha L P, Sax O C, Kowalewski K A, Bains S S, Dubin J, Herzenberg J E, et al. Chronic knee pain following infrapatellar/suprapatellar magnetic intramedullary lengthening nails versus external fixators in limb length discrepancy: a retrospective review. J Orthop 2023; 51:7-11. doi: 10.1016/j.jor.2023.11.071. 38299066 PMC10825912

[5] Kirkham J J, Williamson P. Core outcome sets in medical research. BMJ Medicine 2022; 1(1): e000284. doi: 10.1136/bmjmed-2022-000284. 36936568 PMC9951367

[6] Williamson P R, Altman D G, Bagley H, Barnes K L, Blazeby J M, Brookes S T, et al. The COMET Handbook: version 1.0. Trials 2017; 18(Suppl 3): 280. doi: 10.1186/s13063-017-1978-4. 28681707 PMC5499094

[7] Kim S J, Pierce W, Sabharwal S. The etiology of short stature affects the clinical outcome of lower limb lengthening using external fixation: a systematic review of 18 trials involving 547 patients. Acta Orthop 2014; 85(2): 181. doi: 10.3109/17453674.2014.899856. 24650027 PMC3967262

[8] Frost M W, Rahbek O, Traerup J, Ceccotti A A, Kold S. Systematic review of complications with externally controlled motorized intramedullary bone lengthening nails (FITBONE and PRECICE) in 983 segments. Acta Orthop 2021; 92(1): 120-7. doi: 10.1080/17453674.2020.1835321. 33106069 PMC7919879

[9] Sheridan G A, Fragomen A T, Rozbruch S R. Integrated limb lengthening is superior to classical limb lengthening: a systematic review and meta-analysis of the literature. J Am Acad Orthop Surg Glob Res Rev. 2020; 4(6). doi: 10.5435/JAAOSGLOBAL-D-20-00054 PMC7322778 32656477

[10] Axelrod D, Rubinger L, Shah A, Guy P, Johal H. How should we lengthen post-traumatic limb defects? A systematic review and comparison of motorized lengthening systems, combined internal and external fixation and external fixation alone. Eur J Orthop SurgTraumatol 2021; 31(6): 1015-22. doi: 10.1007/s00590-020-02831-y. 33222112

[11] Frost M W, Rahbek O, Iobst C, Bafor A, Duncan M, Kold S. Complications and risk factors of intramedullary bone lengthening nails: a retrospective multicenter cohort study of 314 FITBONE and PRECICE nails. Acta Orthop 2023; 94: 51. doi: 10.2340/17453674.2023.8479. 36807707 PMC9940487

[12] Wang A, Menon R, Li T, Harris L, Harris I A, Naylor J, et al. Has the degree of outcome reporting bias in surgical randomized trials changed? A meta-regression analysis. ANZ J Surg 2023; 93(1-2): 76-82. doi: 10.1111/ans.18273. 36655339

[13] Menon R, Wang A, Chamberlain K, Harris L, Li T, Harris I A, et al. Has the reporting of patient-important outcomes improved in surgical trials? A meta-epidemiological study. ANZ J Surg 2021; 91(10): 2014-20. doi: 10.1111/ans.16922. 33982387

[14] Saddawi-Konefka D, Kim H M, Chung K C. A systematic review of outcomes and complications of reconstruction and amputation for Type IIIB and IIIC fractures of the tibia. Plast Reconstr Surg 2008; 122(6): 1796-1805. doi: 10.1097/PRS.0b013e31818d69c3. 19050533 PMC4410276

[15] Tricco A C, Lillie E, Zarin W, O’Brien K K, Colquhoun H, Levac D, et al. PRISMA extension for scoping reviews (PRISMA-ScR): checklist and explanation. Ann Intern Med 2018; 169(7): 467-73. doi: 10.7326/M18-0850. 30178033

[16] Peters M D, Godfrey C, McInerney P, Munn Z, Tricco A C, Khalil H. Scoping reviews. JBI Manual for Evidence Synthesis. JBI; 2024. doi: 10.46658/JBIMES-24-09. 33038124

[17] Ouzzani M, Hammady H, Fedorowicz Z, Elmagarmid A. Rayyan—a web and mobile app for systematic reviews. Syst Rev 2016; 5(1): 210. doi: 10.1186/s13643-016-0384-4. 27919275 PMC5139140

[18] Harris P A, Taylor R, Minor B L, Elliott V, Fernandez M, O’Neal L, et al. The REDCap consortium: building an international community of software platform partners. J Biomed Inform 2019; 95: 103208. doi: 10.1016/j.jbi.2019.103208. 31078660 PMC7254481

[19] Harris P A, Taylor R, Thielke R, Payne J, Gonzalez N, Conde J G. Research electronic data capture (REDCap): a metadata-driven methodology and workflow process for providing translational research informatics support. J Biomed Inform 2009; 42(2): 377-81. doi: 10.1016/j.jbi.2008.08.010. 18929686 PMC2700030

[20] Dodd S, Clarke M, Becker L, Mavergames C, Fish R, Williamson P R. A taxonomy has been developed for outcomes in medical research to help improve knowledge discovery. J Clin Epidemiol 2018; 96: 84-92. doi: 10.1016/j.jclinepi.2017.12.020. 29288712 PMC5854263

[21] Boers M, Kirwan J R, Wells G, Beaton D, Gossec L, D’Agostino M A, et al. Developing core outcome measurement sets for clinical trials: OMERACT filter 2.0. J Clin Epidemiol 2014; 67(7): 745-53. doi: 10.1016/j.jclinepi.2013.11.013. 24582946

[22] International Classification of Functioning, Disability and Health (ICF) [Internet]. [cited 2022 Sep 29]. Available from: https://icd.who.int/dev11/l-icf/en#

[23] Beaton D, Maxwell L, Grosskleg S, Shea B, Tugwell B. Chapter 4: Developing core domain sets [Internet]. The OMERACT Handbook Version 21 [updated April 2021] OMERACT. [cited 2024 Jun 26]. Available from: https://omeract.org/handbook/

[24] Page M J, Huang H, Verhagen A P, Gagnier J J, Buchbinder R. Outcome reporting in randomized trials for shoulder disorders: literature review to inform the development of a core outcome set. Arthritis Care Res (Hoboken) 2018; 70(2): 252-9. doi: 10.1002/acr.23254. 28388821

[25] Flourish | Data Visualization & Storytelling [Internet]. [cited 2024 Jul 1]. Available from: https://app.flourish.studio/login

[26] Aquilina A L, Claireaux H, Aquilina C O, Tutton E, Fitzpatrick R, Costa M L, et al. What outcomes have been reported on patients following open lower limb fracture, and how have they been measured? Bone Joint Res 2023; 12(2): 138-46. doi: 10.1302/2046-3758.122.BJR-2022-0116.R1. 37051811 PMC10003018

[27] Kottner J, Beaton D, Clarke M, Dodd S, Kirkham J, Lange T, et al. Core outcome set developers should consider and specify the level of granularity of outcome domains. J Clin Epidemiol 2024; 169. doi: 10.1016/j.jclinepi.2024.111307. 38428539

[28] Yalcinkaya A, Rahbek O, Tirta M, Rathleff M S, Iobst C, Kold S. Outcomes and outcome measures to inform the development of a core outcome set for lower-limb lengthening surgery: a scoping review protocol. Open Science Framework; 2022. doi: 10.17605/OSF.IO/A2D65. PMC11605704 39611437

[29] Haddaway N R, Page M J, Pritchard C C, McGuinness L A. PRISMA2020: An R package and Shiny app for producing PRISMA 2020-compliant flow diagrams, with interactivity for optimised digital transparency and open synthesis. Campbell Syst Rev 2022; 18(2): e1230. doi: 10.1002/cl2.1230. 36911350 PMC8958186

[30] Machielsen A J H M, Iqbal N, Kimman M L, Sahnan K, Adegbola S O, Kane G, et al. Heterogeneity in outcome selection, definition and measurement in studies assessing the treatment of cryptoglandular anal fistula: findings from a systematic review. Tech Coloproctol 2021; 25(7): 761. doi: 10.1007/s10151-021-02452-5. 33963945 PMC8187216

[31] Paley D, Catagni M A, Argnani F, Villa A, Battista Benedetti G, Cattaneo R. Ilizarov treatment of tibial nonunions with bone loss. Clin Orthop Relat Res 1989; 241(241): 146-65. doi: 10.1097/00003086-198904000-00017. 2924458

[32] Fabricant P, Borst E, Green S, Marx R, Fragomen A, Rozbruch S R. Validation of a modified Scoliosis Research Society instrument for patients with limb deformity: The limb deformity-Scoliosis Research Society (LD-SRS) score. J Limb Lengthening Reconstr. 2016; 2(2): 86. doi: 10.4103/2455-3719.190710.

[33] Varni J W, Seid M, Kurtin P S. PedsQLTM 4.0: Reliability and validity of the Pediatric Quality of Life InventoryTM Version 4.0 generic core scales in healthy and patient populations. Med Care 2001; 39(8). doi: 10.1097/00005650-200108000-00006. 11468499

[34] Iliadis A D, Palloni V, Wright J, Goodier D, Calder P. Pediatric lower limb lengthening using the PRECICE nail: our experience with 50 cases. J Pediatr Orthop 2021; 41(1): e44-9. doi: 10.1097/BPO.0000000000001672 32947442

[35] Paley D. Problems, obstacles, and complications of limb lengthening by the Ilizarov technique. Clin Orthop Relat Res 1990; 250(1): 81-104. doi: 10.1097/00003086-199001000-00011. 2403498

[36] Lascombes P, Popkov D, Huber H, Haumont T, Journeau P. Classification of complications after progressive long bone lengthening: proposal for a new classification. Orthop Traumatol Surg Res 2012; 98(6): 629-37. doi: 10.1016/j.otsr.2012.05.010. 22981643

[37] Swiontkowski M F, Buckwalter J A, Keller R B, Haralson. The outcomes movement in orthopaedic surgery: where we are and where we should go. J Bone Joint Surg Am 1999; 81(5): 732-40. doi: 10.2106/00004623-199905000-00016. 10360703

[38] Antonios T, Barker A, Ibrahim I, Scarsbrook C, Smitham P J, David Goodier W, et al. A systematic review of patient-reported outcome measures used in circular frame fixation. Strategies Trauma Limb Reconstr 2019; 14(1): 34. doi: 10.5005/jp-journals-10080-1413. 32559266 PMC7001598

[39] Kim S J, Balce G C, Agashe M V, Song S H, Song H R. Is bilateral lower limb lengthening appropriate for achondroplasia?: midterm analysis of the complications and quality of life. Clin Orthop Relat Res 2012; 470(2): 616-21. doi: 10.1007/s11999-011-1983-y. 21785895 PMC3254769

[40] Bafor A, Iobst C A. what’s new in limb lengthening and deformity correction. J Bone Joint Surg 2022; 104(16): 1419-25. doi: 10.2106/JBJS.22.00398. 35703147

[41] Modin M, Ramos T, Stomberg M W. Postoperative impact of daily life after primary treatment of proximal/distal tibia fracture with Ilizarov external fixation. J Clin Nurs 2009; 18(24): 3498-506. doi: 10.1111/j.1365-2702.2009.02859.x. 19732246

[42] Hrutkay J M, Eilert R E. Operative lengthening of the lower extremity and associated psychological aspects: the Children’s Hospital experience. J Pediatr Orthop 1990; 10(3): 373-7. doi: 10.1097/01241398-199005000-00015. 2355082

[43] World Bank Country and Lending Groups—World Bank Data Help Desk [Internet]. [cited 2024 Jun 26]. Available from: https://datahelpdesk.worldbank.org/knowledgebase/articles/906519-world-bank-country-and-lending-groups

[44] Hafez M, Nicolaou N, Offiah A, Obasohan P, Dixon S, Giles S, et al. How much does paediatric femoral lengthening cost? A cost comparison between magnetic lengthening nails and external fixators. Strategies Trauma Limb Reconstr 2023; 18(1): 16. doi: 10.5005/jp-journals-10080-1573. 38033930 PMC10682557

