# Supplementary material for: Outcomes and outcome measurement instruments in lower-limb lengthening surgery: a scoping review to inform core outcome set development
Source: Acta Orthop. 2024 Nov 29;95:715–22. doi: 10.2340/17453674.2024.42488 (PMC11605704; doi:10.2340/17453674.2024.42488)
Supplement: Supplementary file 1 [file ActaO-95-42488-s1.pdf]

Appendix 1. Search history

Databases and results

Database	Interface	Result	Date
PubMed	NLM	2,658	23.09.2022
Embase	Elsevier	2,982	29.09.2022
Cochrane Library	Wiley	86	22.09.2022
Web of Science	Clarivate	1,615	29.09.2022
Scopus	Elsevier	4,355	29.09.2022
All<br>÷ duplicates with EndNote		11,696<br>5,039	

Update February 2024

Database	Interface	Result	Date
PubMed	NLM	118	12.02.2024
Embase	Elsevier	228	12.02.2024
Cochrane Library	Wiley	6	12.02.2024
Web of Science	Clarivate	132	12.02.2024
Scopus	Elsevier	222	12.02.2024
All<br>÷ duplicates with EndNote		706<br>269	

Update May 2024

Database	Interface	Result	Date
PubMed	NLM	22	22.05.2024
Embase	Elsevier	38	22.05.2024
Cochrane Library	Wiley	0	22.05.2024
Web of Science	Clarivate	22	22.05.2024
Scopus	Elsevier	50	22.05.2024
All<br>÷ duplicates with EndNote		132<br>46	

PubMed

Search	Query	Results
#27	Search: ("Bone Lengthening"[Mesh:NoExp] OR bone lengthening[Text Word] OR limb lengthening[Text Word] OR leg lengthening[Text Word] OR femur lengthening[Text Word] OR femoral lengthening[Text Word] OR tibial lengthening[Text Word] OR limb extensi*[Text Word] OR limb equali*[Text Word]) AND ("Lower Extremity"[Mesh:NoExp] OR "Leg"[Mesh] OR "Thigh"[Mesh] OR "Bones of Lower Extremity"[Mesh:NoExp] OR "Femur"[Mesh] OR "Leg Bones"[Mesh:NoExp] OR "Fibula"[Mesh] OR "Tibia"[Mesh] OR lower extremit*[Text Word] OR lower limb*[Text Word] OR femur[Text Word] OR femoral[Text Word] OR leg[Text Word] OR legs[Text Word] OR tibia*[Text Word]	2,658

	OR fibula*[Text Word] OR calf[Text Word] OR calves[Text Word] OR shin*[Text Word] OR thigh*[Text Word]) Sort by: Most Recent	
#26	Search: "Lower Extremity"[Mesh:NoExp] OR "Leg"[Mesh] OR "Thigh"[Mesh] OR "Bones of Lower Extremity"[Mesh:NoExp] OR "Femur"[Mesh] OR "Leg Bones"[Mesh:NoExp] OR "Fibula"[Mesh] OR "Tibia"[Mesh] OR lower extremit*[Text Word] OR lower limb*[Text Word] OR femur[Text Word] OR femoral[Text Word] OR leg[Text Word] OR legs[Text Word] OR tibia*[Text Word] OR fibula*[Text Word] OR calf[Text Word] OR calves[Text Word] OR shin*[Text Word] OR thigh*[Text Word] Sort by: Most Recent	644,567
#25	Search: calf[Text Word] OR calves[Text Word] OR shin*[Text Word] OR thigh*[Text Word] Sort by: Most Recent	106,445
#24	Search: tibia*[Text Word] OR fibula*[Text Word] Sort by: Most Recent	130,357
#23	Search: leg[Text Word] OR legs[Text Word] Sort by: Most Recent	186,877
#22	Search: femur[Text Word] OR femoral[Text Word] Sort by: Most Recent	224,768
#21	Search: lower limb*[Text Word] Sort by: Most Recent	60,001
#20	Search: lower extremit*[Text Word] Sort by: Most Recent	78,177
#19	Search: "Leg Bones"[Mesh:NoExp] Sort by: Most Recent	584
#18	Search: "Bones of Lower Extremity"[Mesh:NoExp] Sort by: Most Recent	609
#17	Search: "Tibia"[Mesh] Sort by: Most Recent	38,407
#16	Search: "Fibula"[Mesh] Sort by: Most Recent	9,507
#15	Search: "Femur"[Mesh] Sort by: Most Recent	60,313
#14	Search: "Thigh"[Mesh] Sort by: Most Recent	13,541
#13	Search: "Leg"[Mesh] Sort by: Most Recent	65,693
#12	Search: "Lower Extremity"[Mesh:NoExp] Sort by: Most Recent	24,160
#11	Search: "Bone Lengthening"[Mesh:NoExp] OR bone lengthening[Text Word] OR limb lengthening[Text Word] OR leg lengthening[Text Word] OR femur lengthening[Text Word] OR femoral lengthening[Text Word] OR tibial lengthening[Text Word] OR limb extensi*[Text Word] OR limb equali*[Text Word] Sort by: Most Recent	3,875
#10	Search: limb equali*[Text Word] Sort by: Most Recent	17
#9	Search: limb extensi*[Text Word] Sort by: Most Recent	267
#8	Search: tibial lengthening[Text Word] Sort by: Most Recent	314
#7	Search: femoral lengthening[Text Word] Sort by: Most Recent	266
#6	Search: femur lengthening[Text Word] Sort by: Most Recent	34
#5	Search: leg lengthening[Text Word] Sort by: Most Recent	450
#4	Search: limb lengthening[Text Word] Sort by: Most Recent	1,091
#3	Search: bone lengthening[Text Word] Sort by: Most Recent	2,635
#2	Search: "Bone Lengthening"[Mesh:NoExp] Sort by: Most Recent	2,318

Embase

No.	Query	Results
#15	#13 NOT #14	2,982
#14	#13 AND ('conference abstract'/it OR 'conference paper'/it OR 'conference review'/it)	315
#13	#4 AND #12	3297
#12	#5 OR #6 OR #7 OR #8 OR #9 OR #10 OR #11	105,1074
#11	calf:ti,ab,kw OR calves:ti,ab,kw OR shin*:ti,ab,kw OR thigh*:ti,ab,kw	141,703
#10	tibia*:ti,ab,kw OR fibula*:ti,ab,kw	134,814
#9	leg:ti,ab,kw OR legs:ti,ab,kw	180,167
#8	femur:ti,ab,kw OR femoral:ti,ab,kw	249,708
#7	(lower NEAR/1 (limb* OR extremit*)):ti,ab,kw	174,780

#6	'leg bone'/de OR 'femur'/exp OR 'fibula'/exp OR 'tibia'/exp	152,184
#5	'lower limb'/exp	489,109
#4	#1 OR #2 OR #3	5,004
#3	(limb NEAR/1 (equali* OR extensi*)):ti,ab,kw	628
#2	((bone OR limb OR leg OR femur OR femoral OR tibial) NEAR/1 lengthening):ti,ab,kw	2,585
#1	'leg lengthening'/de	3,356

Cochrane

ID	Search	Hits
#1	MeSH descriptor: [Bone Lengthening] explode all trees	102
#2	((bone OR limb OR leg OR femur OR femoral OR tibial) NEAR/1 lengthening):ti,ab,kw	60
#3	(limb NEAR/1 (extensi* OR equali*)):ti,ab,kw	19
#4	{OR #1-#3}	159
#5	MeSH descriptor: [Lower Extremity] this term only	1,502
#6	MeSH descriptor: [Leg] explode all trees	3,007
#7	MeSH descriptor: [Thigh] explode all trees	529
#8	MeSH descriptor: [Femur] explode all trees	1,373
#9	MeSH descriptor: [Fibula] explode all trees	81
#10	MeSH descriptor: [Tibia] explode all trees	663
#11	MeSH descriptor: [Bones of Lower Extremity] this term only	7
#12	MeSH descriptor: [Leg Bones] this term only	15
#13	(lower NEXT limb*):ti,ab,kw	12,110
#14	(lower NEXT extremit*):ti,ab,kw	9,160
#15	(femur OR femoral OR leg OR legs OR tibia* OR fibula*):ti,ab,kw	47,306
#16	(calf OR calves OR shin* OR thigh*):ti,ab,kw	8,672
#17	{OR #5-#16}	64,989
#18	#4 AND #17	86

Web of Science

#	Searches	Hits
#1	(Bone OR limb OR leg OR femur OR femoral OR tibial) NEAR/1 lengthening (Topic)	2,120
#2	TS=(limb NEAR/1 (extension* OR equaliz*))	510
#3	#1 OR #2	2,610
#4	lower NEAR/1 (extremit* OR limb*) (Topic)	115,981
#5	leg OR legs OR femur OR femoral OR tibia* OR fibula* OR calf OR calves OR shin* OR thigh* (Topic)	569,663
#6	#4 OR #5	652,318
#7	#3 AND #6	1,739
#8	#3 AND #6 and Meeting Abstract or Proceeding Paper (Exclude – Document Types)	1,615

Scopus

#	Searches	Hits

#8	((TITLE-ABS-KEY ("bone lengthening" OR "limb lengthening" OR "leg lengthening" OR "femur lengthening" OR "femoral lengthening" OR "tibial lengthening")) OR (TITLE-ABS-KEY ("limb extension*" OR "limb equaliz*"))) AND ((TITLE-ABS-KEY ("lower extremit*" OR "lower limb*")) OR (TITLE-ABS-KEY (leg OR legs OR femur OR femoral OR tibia* OR fibula* OR calf OR calves OR shin* OR thigh*))) AND (EXCLUDE (DOCTYPE , "cp") OR EXCLUDE (DOCTYPE , "ch") OR EXCLUDE (DOCTYPE , "bk"))	4,355
#7	((TITLE-ABS-KEY ("bone lengthening" OR "limb lengthening" OR "leg lengthening" OR "femur lengthening" OR "femoral lengthening" OR "tibial lengthening")) OR (TITLE-ABS-KEY ("limb extension*" OR "limb equaliz*"))) AND ((TITLE-ABS-KEY ("lower extremit*" OR "lower limb*")) OR (TITLE-ABS-KEY (leg OR legs OR femur OR femoral OR tibia* OR fibula* OR calf OR calves OR shin* OR thigh*)))	4,554
#6	(TITLE-ABS-KEY ("lower extremit*" OR "lower limb*")) OR (TITLE-ABS-KEY (leg OR legs OR femur OR femoral OR tibia* OR fibula* OR calf OR calves OR shin* OR thigh*))	975,157
#5	TITLE-ABS-KEY (leg OR legs OR femur OR femoral OR tibia* OR fibula* OR calf OR calves OR shin* OR thigh*)	892,211
#4	TITLE-ABS-KEY ("lower extremit*" OR "lower limb*")	163,077
#3	(TITLE-ABS-KEY ("bone lengthening" OR "limb lengthening" OR "leg lengthening" OR "femur lengthening" OR "femoral lengthening" OR "tibial lengthening")) OR (TITLE-ABS-KEY ("limb extension*" OR "limb equaliz*"))	5,413
#2	TITLE-ABS-KEY ("limb extension*" OR "limb equaliz*")	419
#1	TITLE-ABS-KEY ("bone lengthening" OR "limb lengthening" OR "leg lengthening" OR "femur lengthening" OR "femoral lengthening" OR "tibial lengthening")	5,005
