# Supplementary material for: Outcomes and outcome measurement instruments in lower-limb lengthening surgery: a scoping review to inform core outcome set development
Source: Acta Orthop. 2024 Nov 29;95:715–22. doi: 10.2340/17453674.2024.42488 (PMC11605704; doi:10.2340/17453674.2024.42488)
Supplement: Supplementary file 2 [file ActaO-95-42488-s2.pdf]

Appendix 2.1. Data extraction form on Excel

Title	Publication Year	Verbatim Outcomes	Primary/ Secondary outcome	CORE AREAS	OUTCOME DOMAINS	OUTCOME HEADINGS	Subheadings

Title	Year	Outcome/ Domain	Definitions - Outcome Measurement Instruments (OMIs)	Outcome Heading	Outcome Domain

OMI - Composite Outcomes	Facets within OMIs	Definition of OMI (from the article or the reference)
