# Supplementary material for: Outcomes and outcome measurement instruments in lower-limb lengthening surgery: a scoping review to inform core outcome set development
Source: Acta Orthop. 2024 Nov 29;95:715–22. doi: 10.2340/17453674.2024.42488 (PMC11605704; doi:10.2340/17453674.2024.42488)
Supplement: Supplementary file 3 [file ActaO-95-42488-s3.pdf]

COS

Record ID	________________________
Article	________________________
Publication year	◯ 2024<br>◯ 2023<br>◯ 2022<br>◯ 2021<br>◯ 2020<br>◯ 2019<br>◯ 2018<br>◯ 2017<br>◯ 2016<br>◯ 2015<br>◯ 2014
Intervention type	☐ PRECICE ®<br>☐ PRECICE (P1) ®<br>☐ PRECICE (P2) ®<br>☐ Extramedullary use of PRECICE ®<br>☐ PRECICE plate ® (magnetic expandable plate)<br>☐ STRYDE nail ® (STRYDE, or PRECICE STRYDE)<br>☐ FITBONE ®<br>☐ ISKD ® nail<br>☐ Internal lengthening nail<br>☐ Magnetic internal lengthening nail<br>☐ Mechanical internal lengthening nail<br>☐ Monolateral external fixators (Orthofix, MRS-Modular Rail System, limb reconstruction system, monorail external fixator, etc.)<br>☐ Circular external fixator - Ilizarov<br>☐ Circular external fixator - Classic-type<br>☐ Circular external fixator - Hexapod<br>☐ Circular external fixator - Computer-controlled hexapod<br>☐ Circular external fixator - TSF (Taylor Spatial Frame)<br>☐ Lengthening and then nailing (LATN)<br>☐ Other
Other	________________________
Intervention site	☐ Femur<br>☐ Tibia

Journal name	◯ Acta Orthopaedica<br>◯ Acta Orthopaedica Belgica<br>◯ Archives of Orthopaedic and Trauma Surgery<br>◯ BMC Musculoskeletal Disorders<br>◯ BMC Surgery<br>◯ Children (MDPI)<br>◯ Clinical Orthopaedics and Related Research<br>◯ Indian Journal of Orthopaedics<br>◯ Injury<br>◯ International Orthopaedics<br>◯ JBJS (The Journal of Bone and Joint Surgery)<br>◯ JBJS Case Connector<br>◯ Journal of Children's Orthopaedics<br>◯ Journal of Clinical Medicine (MDPI)<br>◯ Journal of Limb Lengthening & Reconstruction (JLLR)<br>◯ Journal of Orthopaedic Science<br>◯ Journal of Orthopaedic Surgery<br>◯ Journal of Orthopaedics<br>◯ Journal of Orthopaedics Trauma<br>◯ Journal of Pediatric Orthopaedics<br>◯ Journal of Pediatric Orthopaedics B<br>◯ Ortopedia Traumatologia Rehabilitacja<br>◯ Orthopaedic Surgery<br>◯ Orthopaedics & Traumatology: Surgery & Research<br>◯ Orthopedics<br>◯ Strategies in Trauma and Limb Reconstruction<br>◯ Techniques in Orthopaedics<br>◯ THE BONE & JOINT JOURNAL<br>◯ Other
Other	______________________________

Location

◯ Afghanistan
◯ Albania
◯ Algeria
◯ Andorra
◯ Angola
◯ Antigua and Barbuda
◯ Argentina
◯ Armenia
◯ Aruba
◯ Australia
◯ Austria
◯ Azerbaijan
◯ Bahrain
◯ Bangladesh
◯ Barbados
◯ Belarus
◯ Belgium
◯ Belize
◯ Benin
◯ Bhutan
◯ Bolivia
◯ Bosnia and Herzegovina
◯ Botswana
◯ Brazil
◯ Brunei
◯ Bulgaria
◯ Burkina Faso
◯ Burma
◯ Burundi
◯ Cambodia
◯ Cameroon
◯ Canada
◯ Cape Verde
◯ Central African Republic
◯ Chad
◯ Chile
◯ China
◯ Colombia
◯ Comoros
◯ Republic of the
◯ Costa Rica
◯ Cote d'Ivoire
◯ Croatia
◯ Cuba
◯ Curacao
◯ Cyprus
◯ Czech Republic
◯ Denmark
◯ Djibouti
◯ Dominica
◯ Dominican Republic
◯ East Timor (see Timor-Leste)
◯ Ecuador
◯ Egypt
◯ El Salvador
◯ Equatorial Guinea
◯ Eritrea
◯ Estonia
◯ Ethiopia
◯ Fiji
◯ Finland
◯ France
◯ Gabon
◯ Georgia
◯ Germany
◯ Ghana
◯ Greece
◯ Grenada
◯ Guatemala

◯ Guinea
◯ Guinea-Bissau
◯ Guyana
◯ Haiti
◯ Holy See
◯ Honduras
◯ Hong Kong
◯ Hungary
◯ Iceland
◯ India
◯ Indonesia
◯ Iran
◯ Iraq
◯ Ireland
◯ Israel
◯ Italy
◯ Jamaica
◯ Japan
◯ Jordan
◯ Kazakhstan
◯ Kenya
◯ Kiribati
◯ Kosovo
◯ Kuwait
◯ Kyrgyzstan
◯ Laos
◯ Latvia
◯ Lebanon
◯ Lesotho
◯ Liberia
◯ Libya
◯ Liechtenstein
◯ Lithuania
◯ Luxembourg
◯ Macau
◯ Macedonia
◯ Madagascar
◯ Malawi
◯ Malaysia
◯ Maldives
◯ Mali
◯ Malta
◯ Marshall Islands
◯ Mauritania
◯ Mauritius
◯ Mexico
◯ Micronesia
◯ Moldova
◯ Monaco
◯ Mongolia
◯ Montenegro
◯ Morocco
◯ Mozambique
◯ Namibia
◯ Nauru
◯ Nepal
◯ Netherlands
◯ Netherlands Antilles
◯ New Zealand
◯ Nicaragua
◯ Niger
◯ Nigeria
◯ North Korea
◯ Norway
◯ Oman
◯ Pakistan
◯ Palau
◯ Palestinian Territories
◯ Panama
◯ Papua New Guinea
◯ Paraguay

◯ Peru
◯ Philippines
◯ Poland
◯ Portugal
◯ Qatar
◯ Romania
◯ Russia
◯ Rwanda
◯ Saint Kitts and Nevis
◯ Saint Lucia
◯ Saint Vincent and the Grenadines
◯ Samoa
◯ San Marino
◯ Sao Tome and Principe
◯ Saudi Arabia
◯ Senegal
◯ Serbia
◯ Seychelles
◯ Sierra Leone
◯ Singapore
◯ Sint Maarten
◯ Slovakia
◯ Slovenia
◯ Solomon Islands
◯ Somalia
◯ South Africa
◯ South Korea
◯ South Sudan
◯ Spain
◯ Sri Lanka
◯ Sudan
◯ Suriname
◯ Swaziland
◯ Sweden
◯ Switzerland
◯ Syria
◯ Taiwan
◯ Tajikistan
◯ Tanzania
◯ Thailand
◯ Timor-Leste
◯ Togo
◯ Tonga
◯ Trinidad and Tobago
◯ Tunisia
◯ Turkey
◯ Turkmenistan
◯ Tuvalu
◯ Uganda
◯ Ukraine
◯ United Arab Emirates
◯ United Kingdom
◯ United States of America
◯ Uruguay
◯ Uzbekistan
◯ Vanuatu
◯ Venezuela
◯ Vietnam
◯ Yemen
◯ Zambia
◯ Zimbabwe
◯ Other
◯ South Korea

Other	____________________
Study centers	◯ Single-center<br>◯ Multicenter
Study design<br><br>Only choose two types when you are confused	☐ Case report<br>☐ Case series<br>☐ (Database) review (of prospectively collected data)<br>☐ Case-control<br>☐ Cross-sectional<br>☐ Cohort<br>☐ RCT
Study details	◯ Retrospective<br>◯ Prospective<br>◯ Other
Other	____________________
Mean age (years)	____________________
Age range	____________________
Pediatric, adult, mix?<br><br>The paediatric population is defined as 16 years and younger (≤16) at the time of surgery. For both males and females, 16 years is the cut-off age to divide the population into adult and paediatric. Those studies that include both patients ≤16 and >16 years of age are referred to as MIX studies.	◯ Pediatric<br>◯ Adult<br>◯ Mix
Total number of patients	____________________
Number of females	____________________
Number of males	____________________
Follow-up duration (months)	____________________
Are study inclusion-exclusion criteria clearly stated?	◯ Yes<br>◯ No
Is the primary outcome clearly stated?	◯ Yes<br>◯ No
Are secondary outcomes clearly stated?	◯ Yes<br>◯ No
