# Supplementary material for: Outcomes and outcome measurement instruments in lower-limb lengthening surgery: a scoping review to inform core outcome set development
Source: Acta Orthop. 2024 Nov 29;95:715–22. doi: 10.2340/17453674.2024.42488 (PMC11605704; doi:10.2340/17453674.2024.42488)
Supplement: Supplementary file 4 [file ActaO-95-42488-s4.pdf]

Appendix 3. Outcomes

Outcome categorisation and frequency of outcome reporting according to the COMET taxonomy.

Core area	Outcome domains	Outcome headings	Subheadings	Number of verbatim outcomes	Citation
**Adverse events**	**Adverse events**	Complications		138 (99)	(1–99)
		Complications affecting the long-term outcome		3 (3)	(9,33,96)
		Complications requiring unplanned additional surgery		36 (30)	(1,9,11,19,21,25,33,35,37–41,44,50,60,62,64,67,75,79,81,85,89,93,95–97,100,101)
		Hardware or implant-related complications		4 (4)	(21,23,62,93)
		Intraoperative complications		13 (11)	(9,20,23,33,60,93,96,97,101–103)
**Total (Adverse events)**				**194**	
**Life impact**	**Cognitive functioning**	Cognitive functioning		2 (1)	(104)
	**Delivery of care**	Parent satisfaction	Health services	1 (1)	(63)
			Treatment outcome	1 (1)	(63)
		Patient satisfaction	Health services	11 (10)	(9,22,62,73,101,104–108)
			Transportation services	1 (1)	(104)
			Treatment outcome	13 (13)	(19,22,24,29,43,45,50,73,89,106,109–111)
		Tolerance to hardware		2 (2)	(19,37)
		Total number of surgeries		7 (5)	(7,53,54,70,112)
		Total treatment duration		2 (2)	(51,85)
	**Emotional functioning**	Appearance		9 (9)	(24,62,73,101,104,106–108,113)
		Mental health		6 (6)	(11,24,62,65,101,107)
		Mood		3 (2)	(104,107)
		Self-esteem		3 (3)	(22,104,105)
		Negative feelings (harm)		15 (7)	(22,75,83,104,105,114,115)
	**Global quality of life**	Quality of Life		19 (12)	(13,22,65,70,75,76,79,104,105,107,115,116)
	**Personal circumstances**	Financial resources		1 (1)	(104)
		Home environment		1 (1)	(104)
		Physical environment		1 (1)	(104)
	**Physical functioning**	Carrying, moving and handling objects	Lifting and carrying objects	4 (4)	(36,61,107,114)
			Moving objects with lower extremities	2 (1)	(61)
		Changing basic body position	Getting in and out of the car	1 (1)	(36)
			Rolling over in bed	1 (1)	(36)
			Squatting	4 (4)	(36,108,116,117)
		Energy and fatigue		5 (5)	(65,104,107,114,115)

	Full weight bearing		40 (31)	(1,9,18,21,24,25,29–31,33–35,50,58,60,62,66,73,82,92,93,95,97,100,116,118–123)
	Gait pattern		27 (21)	(8,14,15,19,26,28,42,50,54,58,59,73,108,109,113,116,117,124–127)
	Going up and down stairs		11 (9)	(26,36,61,91,107,108,111,116,117)
	Lower limb function		28 (18)	(9,16,24,36,40,52,57,61,62,83,93,101,107,109,114,116,117,128)
	Maintaining body position	Sitting	2 (2)	(36,40)
		Standing	1 (1)	(36)
	Moving around	Jumping	5 (2)	(36,61)
		Moving around outside the home and other buildings	2 (2)	(36,111)
		Moving around within buildings other than home	4 (4)	(36,40,61,114)
		Moving around within the home	3 (2)	(36,87)
		Running	7 (4)	(36,61,63,114)
	Moving around using equipment		13 (12)	(2,29,34,58,93,108,109,116,117,120,129,130)
	Self-care		22 (19)	(8,14,19,22,36,42,50,54,63,73,75,87,104,106,108,113–115,127)
	Sexual functions		1 (1)	(104)
	Sleep functions		5 (4)	(11,104,114,115)
	Sport participation		15 (10)	(36,40,58,61,65,91,107,108,114,116)
	Using transportation	Using human-powered vehicles	2 (1)	(61)
		Using private motorized transportation	1 (1)	(36)
		Using public motorized transportation	1 (1)	(61)
	Walking		34 (27)	(9,14,22,28,34,43,56,58,61,63,75,86,91–93,104,107–111,115,117,119,120,124,130)
**Role functioning**	Carrying out daily routine		14 (12)	(36,42,65,75,107–109,114–116,125,126)
	Work and employment	Acquiring, keeping and terminating a job	12 (9)	(8,36,65,73,91,104,108,116,127)
		Doing housework	2 (2)	(36,115)
		Education life	3 (3)	(36,114,115)
**Social functioning**	Family relationships		1 (1)	(108)
	Informal social relationships		7 (4)	(104,107,108,114)
	Participation in recreation and leisure		6 (6)	(36,65,104,107,108,115)

**Total (Life impact)**			**384**	
**Physiological or clinical**	**Blood and lymphatic system outcomes**	Blood loss (harm)	11 (9)	(3,33,36,62,66,93,97,127,131)
	**Infection and infestation outcomes**	Bone infection (harm)	1 (1)	(87)
		Deep infection (harm)	29 (27)	(4,7,11,14,21–23,37,39,41,42,44,46,54,65,67,81,84,90,91,93,95,101,103,105,127,132)
		Infection (harm)	29 (23)	(8,11,14,19,24,36,42,47,49–51,54,73,76,78,93,96,108,113,126–128,133)
		Intramedullary infection (harm)	3 (3)	(22,48,49)
		Osteomyelitis (harm)	17 (14)	(10,11,15,19,23,32,33,59,60,97,125,132,134,135)
		Pin site infection (harm)	47 (43)	(3,4,8,10,13,16,18,19,22,24,28,33,37–39,41,42,44,48,52,53,57,59,68–70,77–79,82–85,87,88,91,104,114,125,127,131,134,136)
		Septic events (harm)	5 (5)	(9,60,61,67,75)
		Soft tissue infection (harm)	2 (2)	(23,87)
		Superficial or wound infection (harm)	19 (16)	(11,18,19,42,48,51,54,56,65,70,81,86,103,130–132)
		Surgical site infections (harm)	6 (4)	(9,46,89,132)
	**Musculoskeletal and connective tissue outcomes**	Abnormal bone reaction (harm)	73 (14)	(1,7,19,21,25,47,60,72,106,135,137–140)
		Amputation (harm)	3 (3)	(19,39,67)
		Bone healing	236 (105)	(1,2,5,8–15,18–22,24,29–33,36–39,41–43,45,48–55,57–63,65,67–70,73–79,81–98,100–103,105,106,108,109,111–113,116,120,122–128,133–136,139,141–145)
		Cartilage condition	5 (1)	(116)
		Compartment syndrome (harm)	7 (7)	(10,22,42,60,84,85,102)
		Deformity	76 (52)	(4,6,8,13,14,18,19,21–24,27,28,32,35,37,38,41,42,44,50,51,53,54,56,57,59,61,68,70,73,79,83,85,87,88,90–

			93,95,96,101,103,108,110,113,123,125,126,138,145)
	Fractures (harm)	74 (54)	(2–4,7,8,11–14,16,18,22,23,27,28,33,37–42,44,48,51,53,54,56,57,62,65,68–70,73–75,78,81,82,84,85,87,88,92,94,95,97,103,114,123,133,136,145)
	Hardware or implant failure (harm)	133 (62)	(1,5,7,9,14–16,18–21,25,28,30,31,35,37,38,41,44–48,50,51,53–55,57,60–62,64,65,67,71–73,75–77,89,90,93–97,99–103,105,121,123,127,128,133–135)
	Joint instability (harm)	66 (40)	(5,7,14,16,17,22–24,33,34,37–39,42,44,51,52,54,57,60,65–67,69,73,78,82,85,87,95,97,99,103,108,110,116,117,128,131,133)
	Lengthening	456 (132)	(1–19,21–28,30–46,48–50,52–62,64–71,73–82,84–98,100–106,108,109,112–116,119–129,131,133–136,138,139,141–147)
	Lower limb alignment	196 (72)	(5–15,17,18,22–28,32–35,37,38,40–42,45,52,53,56,58,60–62,66,69,70,73–78,82–88,90,93,94,96,97,100,101,103,104,107,108,110,113,119,125,127,128,142,147)
	Muscle strength	7 (6)	(11,17,91,96,109,116)
	Muscle tightness/ stiffness	3 (2)	(51,103)
	Nail removal/retention ratio	9 (9)	(1,6,35,50,67,71,73,103,128)
	Nonunion or nonhealing (harm)	151 (77)	(1,3–5,7–12,14–18,21–25,27,28,31,33,37–48,51–55,57,60,62,63,65,67–69,74–76,78,81–85,87–89,91,93–97,100–

			103,105,113,126,127,130,134)
	Osteoarthritis (harm)	8 (2)	(40,67)
	Osteonecrosis (harm)	5 (5)	(33,60,78,96,97)
	Pain or discomfort	110 (60)	(1,2,7–9,11,13–15,17,19–21,23–25,31–34,39,42,50,54,62,65,67,73,75,79,86,88,89,91,93,95,98,99,101,103,104,106–111,113–117,124–127,132,134,139,148)
	Pelvic position	5 (3)	(50,58,116)
	Range of Motion	215 (83)	(1,2,7–11,14,15,17,19,21–25,27,29,32–34,37–39,42,44,46,47,50–55,57–60,62,63,65–71,73,74,76,79,82–85,87,89–91,93–97,99,101,103,108,109,111,113,114,116,119,125–127,129,131,135,136,144,147)
	Soft tissue complications (harm)	30 (24)	(8,10,14,19,23,27,32,33,42,49,50,54,73,75,79,83,87,94,96,106,108,110,113,126)
	Spinal alignment	10 (3)	(15,46,87)
	Swelling (harm)	5 (5)	(2,51,108,117,132)
	Synovitis (harm)	1 (1)	(116)
	Time in/with hardware	96 (67)	(1,3,4,6–13,16,18–20,23,24,26,28,30,32,36,40–42,48,52,56,57,59,61,66,68,70,73,74,77–79,81–85,87,88,90,92,93,96–98,110,114,119–122,126,127,131,133,134,136–138,141)
	Wound healing	3 (3)	(22,42,89)
	Wound problems (harm)	9 (9)	(5,11,21,48,49,52,70,89,93)
**Nervous system outcomes**	Neurologic deficits (harm)	47 (35)	(3,5,8,11,16,17,19,21–23,33,38,42,45–47,51,53,68,71,78,81,83–85,87,90,95,100,103,111,114,130,135,136)

	**Skin and subcutaneous tissue outcomes**	Skin lesions (harm)		15 (13)	(10,20,22,38,56,81,84–86,91,95,98,127)
	**Vascular outcomes**	Vascular incidents (harm)		29 (24)	(8,9,21–23,33,38,42,46,51,68,71,73,76,78,85,94,96,100,102,105,111,127,135)
**Total (Physiological or clinical)**				**2252**	
**Resource use**	**Economics**	Cost of the treatment		19 (3)	(44,112,115)
	**Hospital**	Length of stay		20 (18)	(9,18–20,33,37,38,44,66,82,86,89,91,97,98,116,148,149)
		Number and duration of outpatient office visits		3 (3)	(44,112,135)
		Operation duration		19 (15)	(3,13,18,33–36,44,62,91,93,97,99,127,131)
		Radiation exposure during surgery		5 (4)	(13,33,93,97)
		Readmission		4 (2)	(82,149)
	**Need for further intervention**	Medications	Analgesia requirements	18 (6)	(29,38,62,66,97,148)
			Antibiotic use	2 (2)	(11,29)
			Dependence on any medication or medical aids	1 (1)	(104)
			Requirement for blood transfusion	5 (3)	(33,93,97)
		Reoperation		9 (9)	(27,33,50,53,62,67,93,113,125)
		Reprogramming		3 (3)	(4,12,13)
	**Societal/carer burden**	Reliance on others		1 (1)	(104)
**Total (Resource use)**				**109**	
**Grand Total**				**2939**	

The table displays both the number of verbatim outcomes listed under each outcome heading and subheading, as well as the number of articles (in parentheses) reporting these outcome headings and subheadings.

For visualization, an interactive map of the outcomes was created using the web application Flourish. (available at https://public.flourish.studio/visualisation/18576652/) (150)

References (Appendix 3):

1. Vogt B, Rupp C, Gosheger G, Eveslage M, Laufer A, Toporowski G, et al. A clinical and radiological matched-pair analysis of patients treated with the PRECICE and STRYDE magnetically driven motorized intramedullary lengthening nails. Bone Joint J [Internet]. 2023 Jan 1 [cited 2024 Jun 30];105-B(1):88–96. Available from: https://pubmed.ncbi.nlm.nih.gov/36587248/
2. Esmat SM, Sulong AF, Awang MS, Oon ZS, Yusof NM, Esmat SM, et al. A Rare Case of Salter-Harris Type I Growth Plate Injury in a Patient Undergoing Limb-Lengthening Procedure. Cureus [Internet]. 2023 Aug 14 [cited 2024 Jun 30];15(8). Available from: https://www.cureus.com/articles/176306-a-rare-case-of-salter-harris-type-i-growth-plate-injury-in-a-patient-undergoing-limb-lengthening-procedure
3. Matsushita M, Kitoh H, Mishima K, Nagata T, Kamiya Y, Kaneko H, et al. A retrospective comparative study of corrective osteotomy for tibial deformities with the multiaxial correction fixator and the circular fixator. J Clin Orthop Trauma [Internet]. 2020 Jul 1 [cited 2024 Jun 30];11(Suppl 4):S621–5. Available from: https://pubmed.ncbi.nlm.nih.gov/32774038/
4. Danışman M, Yılmaz ET, Özdemir E, Tuncay O, Yılmaz G. Accuracy of the hexapod external fixator in treating tibial angular deformities with or without limb length discrepancy: a retrospective study. J Pediatr Orthop B [Internet]. 2023 Nov 1 [cited 2024 Jun 30];32(6):611. Available from: /pmc/articles/PMC10529806/
5. Hammouda A, Szymczuk V, Gesheff M, Mohamed N, Conway J, Standard S, et al. Acute deformity correction and lengthening using the PRECICE magnetic intramedullary lengthening nail. Journal of Limb Lengthening & Reconstruction. 2020;6(1):20.
6. Georgiadis AG, Albright PD, Huser AJ, Novotny SA, Dahl MT. Antegrade femoral lengthening and simultaneous hemiepiphysiodesis for congenital femoral deficiency. J Child Orthop [Internet]. 2024 [cited 2024 Jun 30];18(2):200–7. Available from: https://pubmed.ncbi.nlm.nih.gov/38567040/
7. Laufer A, Frommer A, Gosheger G, Toporowski G, Duedal Rölfing J, Antfang C, et al. Antegrade Intramedullary Femoral Lengthening and Distal Temporary Hemiepiphysiodesis for Combined Correction of Leg Length Discrepancy and Coronal Angular Deformity in Skeletally Immature Patients. Journal of Clinical Medicine 2023, Vol 12, Page 3022 [Internet]. 2023 Apr 21 [cited 2024 Jun 30];12(8):3022. Available from: https://www.mdpi.com/2077-0383/12/8/3022/htm
8. Yushan M, Hamiti Y, Yalikun A, Lu C, Yusufu A. Bifocal femoral lengthening assisted by preoperative 3-dimensional design in the restoration of posttraumatic limb length discrepancy. BMC Surg [Internet]. 2022 Dec 1 [cited 2024 Jun 30];22(1). Available from: /pmc/articles/PMC9238221/
9. Teulières M, Langlais T, Sales de Gauzy J, Rölfing JD, Accadbled F. Bone Lengthening with a Motorized Intramedullary Nail in 34 Patients with Posttraumatic Limb Length Discrepancies. J Clin Med [Internet]. 2021 Jun 1 [cited 2024 Jun 30];10(11). Available from: https://pubmed.ncbi.nlm.nih.gov/34071540/
10. Zak L, Tiefenboeck TM, Wozasek GE. Bone reconstruction: Subjective evaluation and objective analysis based on conventional digital X-rays - a retrospective evaluation. Orthop Traumatol Surg Res [Internet]. 2021 Dec 1 [cited 2024 Jun 30];107(8). Available from: https://pubmed.ncbi.nlm.nih.gov/34583012/
11. Nogueira M, Silva JOF, Paccola AF, Prado S. Can a checklist protocol improve limb lengthening? Journal of Limb Lengthening & Reconstruction. 2022;8(1):63.
12. Roy A, Pesenti S, Chalopin A, Peltier E, Jouve JL, Launay F. Can the TrueLok Hexapod System™ be used to accurately correct lower limb deformity in children? Orthop Traumatol Surg Res [Internet]. 2020 Nov 1 [cited 2024 Jun 30];106(7):1361–6. Available from: https://pubmed.ncbi.nlm.nih.gov/33046433/

13. Messner J, Chhina H, Davidson S, Abad J, Cooper A. Clinical outcomes in pediatric tibial lengthening and deformity correction: a comparison of the Taylor Spatial Frame with the Orthex Hexapod System. J Child Orthop [Internet]. 2021 Apr 4 [cited 2024 Jun 30];15(2):114. Available from: /pmc/articles/PMC8138794/
14. Nasto LA, Coppa V, Riganti S, Ruzzini L, Manfrini M, Campanacci L, et al. Clinical results and complication rates of lower limb lengthening in paediatric patients using the PRECICE 2 intramedullary magnetic nail: a multicentre study. J Pediatr Orthop B [Internet]. 2020 Nov 1 [cited 2024 Jun 30];29(6):611–7. Available from: https://pubmed.ncbi.nlm.nih.gov/31904740/
15. Jardaly A, Gilbert SR. Combined antegrade femur lengthening and distal deformity correction: a case series. J Orthop Surg Res [Internet]. 2021 Dec 1 [cited 2024 Jun 30];16(1). Available from: https://pubmed.ncbi.nlm.nih.gov/33451359/
16. Popkov A, Pietrzak S, Antonov A, Parol T, Lazović M, Podeszwa D, et al. Combined lengthening for acquired leg length discrepancy: Are there advantages of hydroxyapatite-coated intramedullary nails? Orthop Traumatol Surg Res [Internet]. 2023 May 1 [cited 2024 Jun 30];109(3). Available from: https://pubmed.ncbi.nlm.nih.gov/34628087/
17. Wongcharoenwatana J, Hoellwarth JS, Greenstein MD, Reif TJ, Fragomen AT, Rozbruch SR. Comparative fixation devices for preventing migration of the proximal tibiofibular joint during tibial lengthening: a tether versus screw fixation. J Orthop Surg Res [Internet]. 2023 Dec 1 [cited 2024 Jun 30];18(1):1–7. Available from: https://josr-online.biomedcentral.com/articles/10.1186/s13018-023-03771-z
18. Pietrzak S, Grzelecki D, Parol T, Czubak J. Comparison of Intramedullary Magnetic Nail, Monolateral External Distractor, and Spatial External Fixator in Femur Lengthening in Adolescents with Congenital Diseases. J Clin Med [Internet]. 2021 Dec 1 [cited 2024 Jun 30];10(24). Available from: https://pubmed.ncbi.nlm.nih.gov/34945254/
19. Tay KS, Langit M, Fenton C, Grupping R, Muir R, Moulder E, et al. Complex Ankle Fusion With Circular Frames: Factors Influencing Outcomes, Complications, and Patient Satisfaction. Foot Ankle Int [Internet]. 2023 May 1 [cited 2024 Jun 30];44(5):403–14. Available from: https://pubmed.ncbi.nlm.nih.gov/36942403/
20. Ali M, Ali SA, Iqbal Y, Dar UZ, Yousafzai S, Shoaib T. Complications of NA External Fixator in patients who Underwent Tibial Lengthening Surgery. Pakistan Journal of Medical and Health Sciences. 2022 Apr 29;16(4):54–6.
21. Thaller PH, Frankenberg F, Degen N, Soo C, Wolf F, Euler E, et al. Complications and Effectiveness of Intramedullary Limb Lengthening: A Matched Pair Analysis of Two Different Lengthening Nails. Strategies Trauma Limb Reconstr. 2020;15(1):7–12.
22. Nguyen Van L, Le Van D. Complications and functional, psychological outcomes of bilateral tibial lengthening over intramedullary nail: evidence from Vietnam. Int Orthop [Internet]. 2021 Aug 1 [cited 2024 Jun 30];45(8):2007–15. Available from: https://pubmed.ncbi.nlm.nih.gov/33978783/
23. Frost MW, Rahbek O, Iobst C, Bafor A, Duncan M, Kold S. Complications and risk factors of intramedullary bone lengthening nails: a retrospective multicenter cohort study of 314 FITBONE and PRECICE nails. Acta Orthop [Internet]. 2023 [cited 2024 Jun 30];94:51–9. Available from: https://pubmed.ncbi.nlm.nih.gov/36807707/
24. Sagade B, Jagani N, Chaudhary I, Chaudhary M. Congenital Posteromedial Bowing of Tibia: Comparison of Early and Late Lengthening. J Pediatr Orthop [Internet]. 2021 Oct 1 [cited 2024 Jun 30];41(9):E816–22. Available from: https://pubmed.ncbi.nlm.nih.gov/34387229/
25. Radler C, Mindler GT, Stauffer A, Weiß C, Ganger R. Correction of post-traumatic lower-limb discrepancy with Precice intramedullary lengthening nails: a review of 34 adults with an average follow-up of 2 years. Acta Orthop [Internet]. 2022 [cited 2024 Jun 30];93:696. Available from: /pmc/articles/PMC9450255/
26. Kim WJ;, Kim B;, Nho JH;, Kim J;, Hong CH;, Kwon SW;, et al. Crossed Congenital Hemihyperplasia: A Case Report. Surgeries 2022, Vol 3, Pages 242-247 [Internet]. 2022 Aug 23 [cited 2024 Jun 30];3(3):242–7. Available from: https://www.mdpi.com/2673-4095/3/3/26/htm

27. Di Gennaro GL, Gallone G, Martinez Vazquez EA, Marchesini Reggiani L, Racano C, Olivotto E, et al. Deformity progression in congenital posteromedial bowing of the tibia: a report of 44 cases. BMC Musculoskelet Disord [Internet]. 2020 Jul 3 [cited 2024 Jun 30];21(1). Available from: https://pubmed.ncbi.nlm.nih.gov/32620101/
28. Chowdhury JMY, Ahmadi M, Prior CP, Pease F, Messner J, Foster PAL. Distal tibial osteotomy compared to proximal osteotomy for limb lengthening in children. Bone Joint J [Internet]. 2022 Nov 1 [cited 2024 Jun 30];104-B(11):1273–8. Available from: https://pubmed.ncbi.nlm.nih.gov/36317348/
29. Idulhaq M, Santoso A, Mariyanto I, Utomo P. Distraction osteogenesis at the site of previously cystic bone lesion of femur: A case report. Int J Surg Case Rep [Internet]. 2021 Jul 1 [cited 2024 Jun 30];84. Available from: https://pubmed.ncbi.nlm.nih.gov/34216918/
30. Bafor A, Duncan ME, Iobst CA. Early Weight-bearing Accelerates Regenerate Bone Mineralisation: A Pilot Study Comparing Two Post-operative Weight-bearing Protocols Following Intramedullary Limb Lengthening Using the Pixel Value Ratio. Strategies Trauma Limb Reconstr [Internet]. 2022 Sep 1 [cited 2024 Jun 30];17(3):148. Available from: /pmc/articles/PMC9886033/
31. Brinker MR, Amirian A, O'Connor DP, Laughlin MS. Efficacy of PRECICE Nail in Treatment of Adult Patients With Posttraumatic Femoral Leg Length Discrepancy. J Orthop Trauma [Internet]. 2021 Aug 1 [cited 2024 Jun 30];35(8):E304–8. Available from: https://pubmed.ncbi.nlm.nih.gov/33512857/
32. Dossanov B, Trofimchuk V, Lozovoy V, Khmyzov S, Dossanova A, Zhukenov O, et al. Evaluating the results of long tubular bone distraction with an advanced rod monolateral external fixator for achondroplasia. Sci Rep [Internet]. 2021 Dec 1 [cited 2024 Jun 30];11(1). Available from: https://pubmed.ncbi.nlm.nih.gov/34282216/
33. Vogt B, Laufer A, Gosheger G, Toporowski G, Antfang C, Rölfing JD, et al. Evaluation of simultaneous bilateral femoral distraction osteogenesis with antegrade intramedullary lengthening nails in achondroplasia with rhizomelic short stature: a retrospective study of 15 patients with a minimum follow-up of 2 years. Acta Orthop [Internet]. 2024 [cited 2024 Jun 30];95:47. Available from: /pmc/articles/PMC10825798/
34. Schiedel F. Extracorporal noninvasive acute retraction of STRYDE® for continued lengthening in cases with limited nail stroke: a technical less invasive solution to reload the STRYDE®. Arch Orthop Trauma Surg. 2021 Jun 1;141(6):899–905.
35. Dahl MT, Morrison SG, Laine JC, Novotny SA, Georgiadis AG. Extramedullary Motorized Lengthening of the Femur in Young Children. J Pediatr Orthop [Internet]. 2020 Nov 1 [cited 2024 Jun 30];40(10):E978–83. Available from: https://pubmed.ncbi.nlm.nih.gov/32604346/
36. Cheng K, Peng Y, Yan X, Wen X, Ding H. Feasibility of Computer-Aided Design in Limb Lengthening Surgery: Surgical Simulation and Guide Plates. Orthop Surg [Internet]. 2022 [cited 2024 Jun 30];14(9). Available from: https://pubmed.ncbi.nlm.nih.gov/35924698/
37. Hafez M, Nicolaou N, Offiah A, Giles SN, Madan SS, Fernandes JA. Femoral Lengthening in Children-A Comparison Between Magnetic Intramedullary Lengthening Nails and External Fixators. J Pediatr Orthop [Internet]. 2022 Mar 1 [cited 2024 Jun 30];42(3):E290–4. Available from: https://pubmed.ncbi.nlm.nih.gov/34967804/
38. Tillotson LO, Maddock CL, Hanley J, Arseneau GM, Bradley CS, Kelley SP. Femoral lengthening in children: A comparison of motorized intramedullary nailing versus external fixation techniques. Journal of Pediatric Orthopaedics. 2022 May 1;42(5):253–9.
39. Sundar VK, Kannan R, Sharavanan G. FEMORAL LENGTHENING IN CHILDREN: A PROSPECTIVE STUDY. J Cardiovasc Dis Res. 2022;13(6):583–8.
40. Bjørge PA, Tveter AT, Steen H, Gunderson R, Horn J. Femoral lengthening might impair physical function and lead to structural changes in adjacent joints: 10 patients with 27 to 34 years' follow-up. Acta Orthop [Internet]. 2021 [cited 2024 Jun 30];92(3):329–34. Available from: https://pubmed.ncbi.nlm.nih.gov/33410356/

41. Danisman M, Bakircioglu S, Tuncay O, Yilmaz G. Femoral lengthening using limb reconstruction system augmented with only one antegrade flexible nail in children: early results. J Pediatr Orthop B [Internet]. 2023 Nov 1 [cited 2024 Jun 30];32(6):604–10. Available from: https://pubmed.ncbi.nlm.nih.gov/37018750/
42. Van Nguyen L, Van Le D. Functional outcomes and complications of tibial lengthening using unilateral external fixation and then plating. A prospective case series. Annals of Medicine and Surgery [Internet]. 2022 Feb 1 [cited 2024 Jun 30];74:103262. Available from: /pmc/articles/PMC8792419/
43. Makhdom AM, Fragomen AT, Rozbruch SR. Hip-Sparing Equalization Procedures for Leg-Length Discrepancy After Total Hip Arthroplasty: A Retrospective Case Series. HSS J [Internet]. 2020 Dec 1 [cited 2024 Jun 30];16(Suppl 2):400–7. Available from: https://pubmed.ncbi.nlm.nih.gov/33380973/
44. Hafez M, Nicolaou N, Offiah A, Obasohan P, Dixon S, Giles S, et al. How Much Does Paediatric Femoral Lengthening Cost? A Cost Comparison between Magnetic Lengthening Nails and External Fixators. Strategies Trauma Limb Reconstr [Internet]. 2023 Jan 1 [cited 2024 Jun 30];18(1):16. Available from: /pmc/articles/PMC10682557/
45. Rentenberger C, Haider T, Zak L, Wozasek GE. Implant failure after externally magnetic-controlled nail lengthening. Injury [Internet]. 2021 Nov 1 [cited 2024 Jun 30];52(11):3350–4. Available from: https://pubmed.ncbi.nlm.nih.gov/34526237/
46. Egea-Gámez RM, Galán-Olleros M, Alonso-Hernández J, Miranda-Gorozarri C, Martínez-Caballero I, Palazón-Quevedo Á, et al. Improvement of the sagittal alignment of the spine in patients with achondroplasia after subtrochanteric femoral lengthening. Spine Deform [Internet]. 2022 Nov 1 [cited 2024 Jun 30];10(6):1443–52. Available from: https://pubmed.ncbi.nlm.nih.gov/35653063/
47. Sax OC, Hlukha LP, Kowalewski KA, Bains SS, Dubin JA, Herzenberg JE, et al. Incidence of Focal Osteolysis in a Titanium Limb-Lengthening Device: A Comparison to a Stainless-Steel Model. J Orthop Trauma [Internet]. 2023 Dec 1 [cited 2024 Jun 30];37(12):664–8. Available from: https://journals.lww.com/jorthotrauma/fulltext/2023/12000/incidence_of_focal_osteolysis_in_a_titanium.12.aspx
48. Siddiqui NA, Millonig KJ, Mayer BE, Fink JN, McClure PK, Bibbo C. Increased Arthrodesis Rates in Charcot Neuroarthropathy Utilizing Distal Tibial Distraction Osteogenesis Principles. Foot Ankle Spec [Internet]. 2022 Aug 1 [cited 2024 Jun 30];15(4):394–408. Available from: https://pubmed.ncbi.nlm.nih.gov/35506193/
49. Rivera JC, McClure PK, Fragomen AT, Mehta S, Rozbruch SR, Conway JD. Intramedullary Antibiotic Depot Does Not Preclude Successful Intramedullary Lengthening or Compression. J Orthop Trauma [Internet]. 2021 Aug 1 [cited 2024 Jun 30];35(8):E309–14. Available from: https://pubmed.ncbi.nlm.nih.gov/33395176/
50. Zak L, Tiefenboeck TM, Wozasek GE. Intramedullary Bone Lengthening Following Preceding Hip Surgery—A Case Series. Journal of Clinical Medicine 2020, Vol 9, Page 4104 [Internet]. 2020 Dec 19 [cited 2024 Jun 30];9(12):4104. Available from: https://www.mdpi.com/2077-0383/9/12/4104/htm
51. Depaoli A, Magnani M, Casamenti A, Cerasoli T, Ramella M, Menozzi GC, et al. Is the High Healing Index a Complication of Progressive Long Bone Lengthening? Observations from a Cohort of 178 Children Treated with Circular External Fixation for Lower Limb Length Discrepancy. Children (Basel) [Internet]. 2023 Oct 1 [cited 2024 Jun 30];10(10). Available from: https://pubmed.ncbi.nlm.nih.gov/37892249/
52. Ghaly HM, Megahed AHAH, Elmehy EAF, Hegazy MH. Leg lengthening and deformity correction in fibular hemimelia using ring external fixator. Int J Health Sci (Qassim). 2022 Jul 9;3406–25.
53. Johari AN, Anjum R. Lengthening in congenital posteromedial bowing of tibia: a follow-up series at skeletal maturity. Int Orthop [Internet]. 2024 Jun 1 [cited 2024 Jun 30];48(6):1439–52. Available from: https://pubmed.ncbi.nlm.nih.gov/38594586/

54. Campanacci L, Cevolani L, Focaccia M, Di Gennaro GL, Dozza B, Staals E, et al. Lengthening Patients Previously Treated for Massive Lower Limb Reconstruction for Bone Tumors with the PRECICE 2 Nail. Children 2023, Vol 10, Page 1772 [Internet]. 2023 Oct 31 [cited 2024 Jun 30];10(11):1772. Available from: https://www.mdpi.com/2227-9067/10/11/1772/htm
55. Huser AJ, Hoellwarth JS, Coppa V, Feldman DS, Paley D. Lengthening the Lower Extremities of Children with Ollier's and Maffucci's Enchondromatosis Using Implantable Lengthening Nails. Children (Basel) [Internet]. 2021 Jun 1 [cited 2024 Jun 30];8(6). Available from: https://pubmed.ncbi.nlm.nih.gov/34198529/
56. Lazović M, Leonchuk SS, Ducić S, Imomov SA, Popkov DA. Limb lengthening and deformity correction in patients with severe fibular hemimelia: experience of the children's university hospital in Belgrade. Genij Ortopedii. 2024;30(1):38–45.
57. Popkov A, Pietrzak S, Antonov A, Parol T, Lazovic M, Podeszwa D, et al. Limb Lengthening for Congenital Deficiencies Using External Fixation Combined With Flexible Intramedullary Nailing: A Multicenter Study. J Pediatr Orthop [Internet]. 2021 Jul 1 [cited 2024 Jun 30];41(6):e439–47. Available from: https://pubmed.ncbi.nlm.nih.gov/33782369/
58. Iobst C. Limb lengthening in down syndrome. Journal of Limb Lengthening & Reconstruction [Internet]. 2020 [cited 2024 Jun 30];6(2):153. Available from: https://journals.lww.com/jllr/fulltext/2020/06020/limb_lengthening_in_down_syndrome.11.aspx
59. Goodbody CM, Buksbaum J, Harbison MD, Fragomen AT, Rozbruch SR. Limb Lengthening in Russell-Silver Syndrome: An Update Confirming Safe and Speedy Healing. J Pediatr Orthop [Internet]. 2021 Aug 1 [cited 2024 Jun 30];41(7):428–32. Available from: https://pubmed.ncbi.nlm.nih.gov/33999566/
60. Radler C, Mindler GT, Stauffer A, Weiß C, Ganger R. Limb Lengthening With Precice Intramedullary Lengthening Nails in Children and Adolescents. J Pediatr Orthop [Internet]. 2022 Feb 1 [cited 2024 Jun 30];42(2):E192–200. Available from: https://pubmed.ncbi.nlm.nih.gov/34799542/
61. Popkov A, Aranovich A, Antonov A, Journeau P, Lascombes P, Popkov D. Lower limb lengthening and deformity correction in polyostotic fibrous dysplasia using external fixation and flexible intramedullary nailing. J Orthop [Internet]. 2020 Sep 1 [cited 2024 Jun 30];21:192–8. Available from: https://pubmed.ncbi.nlm.nih.gov/32256003/
62. Vogt B, Lueckingsmeier M, Gosheger G, Laufer A, Toporowski G, Antfang C, et al. Magnetically driven antegrade intramedullary lengthening nails for tibial lengthening. Bone Joint J [Internet]. 2024 Mar 1 [cited 2024 Jun 30];106-B(3):293–302. Available from: https://pubmed.ncbi.nlm.nih.gov/38423115/
63. Borici N, Ezeokoli EU, Ruci J, Olldashi T, Borici N, Ezeokoli EU, et al. Management of Femur and Tibial Leg Length Discrepancies With a Unilateral External Fixator Is Still Viable When More Advanced Techniques and Hardware Are Unavailable or Cost-Prohibitive. Cureus [Internet]. 2022 Jan 7 [cited 2024 Jun 30];14(1). Available from: https://www.cureus.com/articles/80485-management-of-femur-and-tibial-leg-length-discrepancies-with-a-unilateral-external-fixator-is-still-viable-when-more-advanced-techniques-and-hardware-are-unavailable-or-cost-prohibitive
64. Hlukha LP, Alrabai HM, Sax OC, Hammouda AI, Mcclure PK, Herzenberg JE. Mechanical Failures in Magnetic Intramedullary Lengthening Nails. J Bone Joint Surg Am [Internet]. 2023 Jan 18 [cited 2024 Jun 30];105(2):113–27. Available from: https://pubmed.ncbi.nlm.nih.gov/36651888/
65. Lecoanet P, Legallois Y, Ribes C, Lefevre Y, Cadennes A, Fabre T. Medium-term evaluation of leg lengthening by ISKD® intramedullary nail in 28 patients: Should we still use this lengthening system? Orthop Traumatol Surg Res [Internet]. 2020 Nov 1 [cited 2024 Jun 30];106(7):1433–40. Available from: https://pubmed.ncbi.nlm.nih.gov/33087298/
66. Georgiadis AG, Gannon NP, Dahl MT. Motorized Plate Lengthening of the Femur in Children: A Preliminary Report. J Pediatr Orthop [Internet]. 2022 Nov 1 [cited 2024 Jun 30];42(10):E987–93. Available from: https://pubmed.ncbi.nlm.nih.gov/36084628/
67. Harrison W, Laubscher M, Al Saifi S, Abdelaziz A, Barnard AC, Birkholtz F, et al. Multicentre review of intramedullary lengthening nails: A middle-income country perspective. Journal of Limb Lengthening & Reconstruction. 2021;7(2):119.

68. Wu C, Huang Y, Huang P, Mo Y, Wang D, Ning B. New bone formation accelerates during lower limb lengthening and deformity correction in children with Ollier's disease. J Orthop Traumatol [Internet]. 2023 Dec 1 [cited 2024 Jun 30];24(1). Available from: https://pubmed.ncbi.nlm.nih.gov/37524995/
69. Hosny GA, Elsheikh AA. Outcomes of intralesional osteotomy and distraction osteogenesis for limb length equalization in Ollier's disease. J Pediatr Orthop B [Internet]. 2023 Jan 1 [cited 2024 Jun 30];32(1):54–9. Available from: https://pubmed.ncbi.nlm.nih.gov/35861695/
70. Bakircioglu S, Goker B, Yilmaz A, Aksoy T, Aksoy MC, Yilmaz G. Outcomes of Limb Lengthening With Computer-assisted Hexapod External Fixators in the Treatment of Fibular Hemimelia. J Pediatr Orthop [Internet]. 2023 Aug 1 [cited 2024 Jun 30];43(7):E574–82. Available from: https://pubmed.ncbi.nlm.nih.gov/37254033/
71. Dubin JA, Bains SS, Moore M, Assayag MA, McClure PK. Outpatient limb lengthening using magnetic intramedullary nails: A single Institution's experience. J Orthop [Internet]. 2024 Jul 1 [cited 2024 Jun 30];53:163–7. Available from: https://pubmed.ncbi.nlm.nih.gov/38601890/
72. Rölfing JD, Kold S, Nygaard T, Mikuzis M, Brix M, Faergemann C, et al. Pain, osteolysis, and periosteal reaction are associated with the STRYDE limb lengthening nail: a nationwide cross-sectional study. Acta Orthop [Internet]. 2021 [cited 2024 Jun 30];92(4):479–84. Available from: https://pubmed.ncbi.nlm.nih.gov/33757381/
73. Iliadis AD, Palloni V, Wright J, Goodier D, Calder P. Pediatric Lower Limb Lengthening Using the PRECICE Nail: Our Experience With 50 Cases. J Pediatr Orthop [Internet]. 2021 Jan 1 [cited 2024 Jun 30];41(1):e44–9. Available from: https://pubmed.ncbi.nlm.nih.gov/32947442/
74. Uzer G, Balcı Hİ, Aliyev O, Ezici V, Demirkıran CB, Yıldız F, et al. Plate fixation after lengthening with an external fixator in patients with achondroplasia : A comparative study between two centers. Orthopadie (Heidelberg, Germany) [Internet]. 2023 Jan 1 [cited 2024 Jun 30];52(1):48–53. Available from: https://pubmed.ncbi.nlm.nih.gov/36280609/
75. Zuluaga M, Pérsico F, Medina J, Reina F, Jiménez N, Benedetti F. PRECICE nail for the management of posttraumatic bone defects with nonunion or malunion: Experience from a Latin American center. Injury [Internet]. 2023 Nov 1 [cited 2024 Jun 30];54 Suppl 6. Available from: https://pubmed.ncbi.nlm.nih.gov/38143138/
76. Galal S, Shin J, Principe P, Khabyeh-Hasbani N, Mehta R, Hamilton A, et al. STRYDE versus PRECICE magnetic internal lengthening nail for femur lengthening. Arch Orthop Trauma Surg [Internet]. 2022 Dec 1 [cited 2024 Jun 30];142(12):3555–61. Available from: https://pubmed.ncbi.nlm.nih.gov/33983528/
77. Mingazov E, Gvozdev N, Popkov A, Journeau P, Popkov D. Preliminary Results of Bone Lengthening over Telescopic Titanium Intramedullary Rod. Case Rep Orthop [Internet]. 2023 Jan 30 [cited 2024 Jun 30];2023:1–16. Available from: /pmc/articles/PMC9902136/
78. Martí PR, Martí IG, Fontecha CG. Prophylactic Intramedullary Rodding After Femoral Lengthening in Patients With Achondroplasia and Hypochondroplasia. J Pediatr Orthop [Internet]. 2024 Mar 1 [cited 2024 Jun 30];44(3):E249–54. Available from: https://pubmed.ncbi.nlm.nih.gov/38084006/
79. Trofimchuk V, Dossanov B, Lozovoy V, Khmyzov S, Dossanova A, Angelov A, et al. Quality of Life in Children With Achondroplasia Undergoing Paired Limb Lengthening With an External Fixator and Modified Distraction Control: Observational Nonrandomized Study. JMIR Rehabil Assist Technol [Internet]. 2024 Jan 1 [cited 2024 Jun 30];11(1). Available from: https://pubmed.ncbi.nlm.nih.gov/38265860/
80. Georgiadis AG, Nahm NJ, Dahl MT. Re-use of Motorised Intramedullary Limb Lengthening Nails. Strategies Trauma Limb Reconstr [Internet]. 2023 May 1 [cited 2024 Jun 30];18(2):106. Available from: /pmc/articles/PMC10628613/
81. Verdoni F, Giorgino R, Virgilio C, Nannini A, Viganò M, Curci D, et al. Results and complications of bilateral limb lengthening in achondroplasia: a retrospective analysis. Front Pediatr. 2023 Feb 8;11.

82. El-Adly W, El-Gafary K, Khashaba A, Khaled M. Results of Ilizarov external fixator lengthening compared to lengthening and then plating in management of femoral shortening in children. Acta Orthop Belg [Internet]. 2023 Jun 1 [cited 2024 Jun 30];89(2):177–82. Available from: https://pubmed.ncbi.nlm.nih.gov/37924532/
83. Huang Q, Ma T, Ren C, Xu YB, Li M, Wang Q, et al. Shortening and re-lengthening versus bone transport for the treatment of distal tibial periarticular post-traumatic defects. Sci Rep [Internet]. 2022 Dec 1 [cited 2024 Jun 30];12(1). Available from: https://pubmed.ncbi.nlm.nih.gov/36175612/
84. Shabtai L, Jauregui JJ, Herzenberg JE, Gesheff MG, Standard SC, McClure PK. Simultaneous Bilateral Femoral and Tibial Lengthening in Achondroplasia. Children (Basel) [Internet]. 2021 Sep 1 [cited 2024 Jun 30];8(9). Available from: https://pubmed.ncbi.nlm.nih.gov/34572181/
85. Ghaly HM, El-Rosasy MAM, Marei AE, El-Gebaly OA. Simultaneous femoral and tibial lengthening for severe limb length discrepancy in fibular hemimelia. J Orthop Surg Res [Internet]. 2023 Dec 1 [cited 2024 Jun 30];18(1). Available from: https://pubmed.ncbi.nlm.nih.gov/37936235/
86. Paley D, Lamm BM, Robbins C, Sullivan A, Ernst J, Danne E, et al. Simultaneous Limb Lengthening With Ankle Fusion Using an Internal Lengthening Nail. J Foot Ankle Surg [Internet]. 2022 Jul 1 [cited 2024 Jun 30];61(4):e15–20. Available from: https://pubmed.ncbi.nlm.nih.gov/34969598/
87. Leiva-Gea A, Delgado-Rufino FB, Queipo-de-Llano A, Mariscal-Lara J, Lombardo-Torre M, Luna-González F. Staged upper and lower limb lengthening performing bilateral simultaneous surgery of the femur and tibia in achondroplastic patients. Arch Orthop Trauma Surg [Internet]. 2020 Nov 1 [cited 2024 Jun 30];140(11):1665–76. Available from: https://pubmed.ncbi.nlm.nih.gov/32048016/
88. Xu L, Song H, Ren Y, Li M, Meng X, Zhou C, et al. The correction of Ollier's disease induced extremities deformity using distraction osteogenesis by intralesional osteotomy in children patients: a cases series study. J Orthop Translat. 2020 Nov 1;25:66–72.
89. Zak L, Arnhold R, Tiefenboeck TM, Wozasek GE. The influence of advanced age in bone healing after intramedullary limb lengthening. Orthop Traumatol Surg Res [Internet]. 2021 Dec 1 [cited 2024 Jun 30];107(8). Available from: https://pubmed.ncbi.nlm.nih.gov/34536594/
90. Liu KY, Wu KW, Lee CC, Lin SC, Kuo KN, Wang TM. Tibial Lengthening along Submuscular Plate with Simultaneous Acute Tibial Deformity Correction by High-Energy Osteotomy: A Comparative Study. J Clin Med [Internet]. 2022 Sep 1 [cited 2024 Jun 30];11(18). Available from: https://pubmed.ncbi.nlm.nih.gov/36143125/
91. Abdel Hameed HAH, El Barbary HM. TIBIAL LENGTHENING IN PATIENTS WITH ACHONDROPLASIA BY ILIZAROV METHOD. European Journal of Molecular & Clinical Medicine. 2020;7(10):2516–21.
92. Popkov A V., Gorbach ES, Mamedov UF, Stepanov R V. Tibial lengthening over a bioactive degradable intramedullary implant: a case report. Genij Ortopedii. 2023;29(6):645–9.
93. Vogt B, Roedl R, Gosheger G, Toporowski G, Laufer A, Theil C, et al. Tibial lengthening using a retrograde magnetically driven intramedullary lengthening device in 10 patients with preexisting ankle and hindfoot fusion. Acta Orthop [Internet]. 2020 [cited 2024 Jun 30];91(6):761. Available from: /pmc/articles/PMC8023964/
94. Geiger EJ, Geffner AD, Rozbruch SR, Fragomen AT. Treatment of Angular Deformity and Limb Length Discrepancy With a Retrograde Femur Magnetic Intramedullary Nail: A Fixator-assisted, Blocking Screw Technique. J Am Acad Orthop Surg Glob Res Rev [Internet]. 2023 May 18 [cited 2024 Jun 30];7(5). Available from: https://journals.lww.com/jaaosglobal/fulltext/2023/05000/treatment_of_angular_deformity_and_limb_length.18.aspx
95. Alonso-Hernández J, Galán-Olleros M, Miranda-Gorozarri C, Egea-Gámez RM, Palazón-Quevedo Á. Two-stage Bone Lengthening With Reuse of a Single Intramedullary Telescopic Nail in Patients With Achondroplasia. J Pediatr Orthop [Internet]. 2022 Jul 1 [cited 2024 Jun 30];42(6):E616–22. Available from: https://pubmed.ncbi.nlm.nih.gov/35250019/
96. Geffner A, Reif T, Fragomen A, Rozbruch Sr. Use and safety of the precice antegrade femoral nail in pediatric patients. Journal of Limb Lengthening & Reconstruction. 2022;8(1):12.

97. Frommer A, Roedl R, Gosheger G, Niemann M, Turkowski D, Toporowski G, et al. What Are the Potential Benefits and Risks of Using Magnetically Driven Antegrade Intramedullary Lengthening Nails for Femoral Lengthening to Treat Leg Length Discrepancy? Clin Orthop Relat Res [Internet]. 2022 Apr 1 [cited 2024 Jun 30];480(4):790–803. Available from: https://pubmed.ncbi.nlm.nih.gov/34780384/
98. Ali SA, Iqbal Y, Anjum S, Ali M, Yousafzai S, Khan MI. Yield of Tibial Lengthening Surgery using NA External Fixator. Pakistan Journal of Medical & Health Sciences [Internet]. 2022 Apr 29 [cited 2024 Jun 30];16(04):57–57. Available from: https://www.pjmhsonline.com/index.php/pjmhs/article/view/658
99. Abalkhail TB, McClure PK. Removal of a Broken Stainless-Steel Intramedullary Lengthening Stryde Nail: A Case Report and Review of the Literature. JBJS Case Connect [Internet]. 2021 Dec 22 [cited 2024 Jun 30];11(4). Available from: https://journals.lww.com/jbjscc/fulltext/2021/12000/removal_of_a_broken_stainless_steel_intramedullary.82.aspx
100. Robbins C, Paley D. Stryde Weight-bearing Internal Lengthening Nail. Techniques in Orthopaedics. 2020;35(3):201–8.
101. Fragomen A, Falls T, Suh J, Khabyeh-Hasbani N, Rozbruch Sr. Tibial lengthening evolution: Classic ilizarov, lengthening and then nailing, motorized internal lengthening nail. Journal of Limb Lengthening & Reconstruction [Internet]. 2020 [cited 2024 Jun 30];6(1):13. Available from: https://journals.lww.com/jllr/fulltext/2020/06010/tibial_lengthening_evolution__classic_ilizarov,.4.aspx
102. Cosic F, Edwards E. PRECICE intramedullary nail in the treatment of adult leg length discrepancy. Injury [Internet]. 2020 Apr 1 [cited 2024 Jun 30];51(4):1091–6. Available from: https://pubmed.ncbi.nlm.nih.gov/32164952/
103. Wright SE, Goodier WD, Calder P. Regenerate Deformity with the Precice Tibial Nail. Strategies Trauma Limb Reconstr [Internet]. 2020 [cited 2024 Jun 30];15(2):98. Available from: /pmc/articles/PMC7801900/
104. Pawik Ł, Pawik M, Wrzosek Z, Fink-Lwow F, Morasiewicz P. Assessment of the quality of life in patients with varying degrees of equalization of lower limb length discrepancy treated with Ilizarov method. J Orthop Surg Res [Internet]. 2021 Dec 1 [cited 2024 Jun 30];16(1). Available from: https://pubmed.ncbi.nlm.nih.gov/33468173/
105. Havitcioglu H, Gursan O, Isin Y. Cosmetic bilateral leg lengthening using intramedullary nail experience of 9 cases. J Orthop [Internet]. 2020 Jul 1 [cited 2024 Jun 30];20:232–5. Available from: https://pubmed.ncbi.nlm.nih.gov/32055149/
106. Iliadis AD, Wright J, Stoddart MT, Goodier WD, Calder P. Early results from a single centre's experience with the STRYDE nail a cause for concern? Bone and Joint Journal. 2021 Jun 1;103 B(6):1168–72.
107. Heath MR, Shin TJ, Mehta R, Principe PS, MacKie AT, Fragomen A, et al. Patients With Lower Limb Deformity Report Worse Quality of Life Than Control Subjects Regardless of Degree of Deformity. JAAOS Global Research & Reviews [Internet]. 2021 Aug 10 [cited 2024 Jun 30];5(8). Available from: /pmc/articles/PMC8357251/
108. Boero S, Marrè Brunenghi G, Riganti S, Torchia S. Role of proximal tibiofibular fixation in leg lengthening with the Ilizarov method in the achondroplastic patient. J Pediatr Orthop B [Internet]. 2023 Jan 1 [cited 2024 Jun 30];32(1):66–71. Available from: https://pubmed.ncbi.nlm.nih.gov/35635539/
109. Copp J, Magister S, Napora J, Getty P, Sontich J. Dual Magnetically Expandable Intramedullary Nails for Treatment of a Large Bony Defect in a Patient with Sarcoma: A Case Report. Strategies Trauma Limb Reconstr [Internet]. 2022 Sep 1 [cited 2024 Jun 30];17(3):189. Available from: /pmc/articles/PMC9886027/
110. Marta R. Limb lengthening of a Rare Case of Congenital Femoral Deficiency with an Unstable Knee. Journal of Limb Lengthening & Reconstruction [Internet]. 2021 Jul 1 [cited 2024 Jun 30];7(2):141–141. Available from: https://go.gale.com/ps/i.do?p=HRCA&sw=w&issn=24553719&v=2.1&it=r&id=GALE%7CA688542651&sid=googleScholar&linkaccess=fulltext

111. Van Nguyen L. Treatment of 18 cm lower limb length discrepancy using lengthening over nail technique in tibia and lengthening and plating in femur – A case report. Int J Surg Case Rep [Internet]. 2023 Mar 1 [cited 2024 Jun 30];104. Available from: /pmc/articles/PMC10018547/
112. Dvorzhinskiy A, Zhang DT, Fragomen AT, Rozbruch SR. Cost Comparison of Tibial Distraction Osteogenesis Using External Lengthening and Then Nailing vs Internal Magnetic Lengthening Nails. Strategies Trauma Limb Reconstr [Internet]. 2021 Jan 1 [cited 2024 Jun 30];16(1):14. Available from: /pmc/articles/PMC8311750/
113. Assayag M, Buksbaum J, Khabyeh-Hasbani N, Westrich E, Fragomen A, Rozbruch Sr. Psychological and orthopedic outcomes after stature lengthening surgery using intramedullary nails. Journal of Limb Lengthening & Reconstruction. 2020;6(1):28.
114. Batıbay SG, Balcı Hİ, Bayram S, Chodza M, Göksoy Ş, Hürmeydan ÖM, et al. Quality of Life Evaluation Following Limb Lengthening Surgery in Patients with Achondroplasia. Indian J Orthop [Internet]. 2020 Sep 1 [cited 2024 Jun 30];54(Suppl 1):39. Available from: /pmc/articles/PMC7474039/
115. Hafez M, Nicolaou N, Offiah A, Offorha B, Giles S, Madan S, et al. Quality of life of children during distraction osteogenesis: a comparison between intramedullary magnetic lengthening nails and external fixators. Int Orthop [Internet]. 2022 Jun 1 [cited 2024 Jun 30];46(6):1367–73. Available from: https://pubmed.ncbi.nlm.nih.gov/35385976/
116. Krieg AH, Dong C, Schmid MP, Speth BM, Harder D, Donners R. Long-term effects of retrograde approach on the knee after motorized femoral limb lengthening. Acta Orthop [Internet]. 2023 [cited 2024 Jun 30];94:128–34. Available from: https://pubmed.ncbi.nlm.nih.gov/36950849/
117. Hlukha LP, Sax OC, Kowalewski KA, Bains SS, Dubin J, Herzenberg JE, et al. Chronic knee pain following infrapatellar/suprapatellar magnetic intramedullary lengthening nails versus external fixators in limb length discrepancy: A retrospective review. J Orthop [Internet]. 2023 May 1 [cited 2024 Jun 30];51:7–11. Available from: https://pubmed.ncbi.nlm.nih.gov/38299066/
118. Bafor A, Duncan ME, Iobst CA. Evaluating the Utility of the Pixel Value Ratio in the Determination of Time to Full Weight-bearing in Patients Undergoing Intramedullary Limb Lengthening. Strategies Trauma Limb Reconstr [Internet]. 2020 May 1 [cited 2024 Jun 30];15(2):74. Available from: /pmc/articles/PMC7801898/
119. Popkov A, Tverdokhlebov S, Muradisinov S, Popkov D. First Clinical Case of Ilizarov Femur Lengthening over a Bioactive and Degradable Intramedullary Implant. Case Rep Orthop [Internet]. 2023 Dec 7 [cited 2024 Jun 30];2023:1–9. Available from: https://pubmed.ncbi.nlm.nih.gov/38099082/
120. Patil VE, Mankar S, Agrawal P, Patil VE, Mankar S, Agrawal PP. Limb Reconstruction Using the Ilizarov Technique Following Giant Cell Tumour Excision in the Proximal Tibia of a 19-Year-Old Female: A Case Report. Cureus [Internet]. 2024 Apr 1 [cited 2024 Jun 30];16(4). Available from: https://www.cureus.com/articles/236318-limb-reconstruction-using-the-ilizarov-technique-following-giant-cell-tumour-excision-in-the-proximal-tibia-of-a-19-year-old-female-a-case-report
121. Iobst CA, Hatfield DN, Forro SD, Quinnan SM. Magnetically Driven Intramedullary Limb Lengthening in Patients with Pre-existing Implanted Programmable Devices: A Case Series. Strategies Trauma Limb Reconstr [Internet]. 2023 May 1 [cited 2024 Jun 30];18(2):111–6. Available from: https://pubmed.ncbi.nlm.nih.gov/37942430/
122. Iobst CA, Bafor A. Retrograde Extramedullary Lengthening of the Femur Using the PRECICE Nail: Technique and Results. J Pediatr Orthop [Internet]. 2021 Jul 1 [cited 2024 Jun 30];41(6):356–61. Available from: https://pubmed.ncbi.nlm.nih.gov/34096549/
123. Elsheikh AA, Wright J, Stoddart MT, Goodier D, Calder P. Use of the Pixel Value Ratio Following Intramedullary Limb Lengthening: Uncomplicated Full Weight-bearing at Lower Threshold Values. Strategies Trauma Limb Reconstr [Internet]. 2022 Jan 1 [cited 2024 Jun 30];17(1):14–8. Available from: https://pubmed.ncbi.nlm.nih.gov/35734036/

124. Femino JD, Nelson SC, Agulnik M, Zuckerman LM. All-Internal Limb Lengthening with Simultaneous Corrective Osteotomy After Internal Hemipelvectomy: A Case Report. JBJS Case Connect [Internet]. 2022 May 12 [cited 2024 Jun 30];12(2). Available from: https://pubmed.ncbi.nlm.nih.gov/36099533/
125. Gordon JE, Schoenecker PL, Lewis TR, Miller ML. Limb lengthening in the treatment of posteromedial bowing of the tibia. J Child Orthop [Internet]. 2020 Oct 10 [cited 2024 Jun 30];14(5):480. Available from: /pmc/articles/PMC7666791/
126. Dabash S, Zhang DT, Rozbruch SR, Fragomen AT. Limb salvage reconstruction of the lower limb with complex ankle arthrodesis and magnetic internal lengthening nail. Eur J Orthop Surg Traumatol [Internet]. 2024 May 1 [cited 2024 Jun 30];34(4):1877–82. Available from: https://pubmed.ncbi.nlm.nih.gov/38441634/
127. Farsetti P, De Maio F, Potenza V, Efremov K, Marsiolo M, Caterini A, et al. Lower limb lengthening over an intramedullary nail: a long-term follow-up study of 28 cases. J Orthop Traumatol [Internet]. 2019 Dec 1 [cited 2024 Jun 30];20(1). Available from: https://pubmed.ncbi.nlm.nih.gov/31506759/
128. Shannon C, Paley D. Extramedullary Internal Limb Lengthening. Techniques in Orthopaedics [Internet]. 2020 [cited 2024 Jun 30];35(3):195–200. Available from: https://journals.lww.com/techortho/fulltext/2020/09000/extramedullary_internal_limb_lengthening.9.aspx
129. Schrijnemakers O, Lejeune G, Ciornohac J, Stefancu A. Management of neglected post-traumatic ipsilateral distal femoral and proximal tibial epiphysiodeses in adolescent: Case report. Trauma Case Rep [Internet]. 2023 Oct 1 [cited 2024 Jun 30];47. Available from: https://pubmed.ncbi.nlm.nih.gov/37693742/
130. Yadav SS. Fibular hemimelia: reconstruction of difficult cases with tibial lengthening and ankle arthrodesis. Int Orthop [Internet]. 2024 May 7 [cited 2024 Jun 30];1–9. Available from: https://link.springer.com/article/10.1007/s00264-024-06183-8
131. Shu W, Yue C, Zhong H, Tang X. Case report: Single-session double-Ilizarov lengthening technique in the treatment of a child with congenital fibular deficiency. Front Pediatr [Internet]. 2022 Jul 29 [cited 2024 Jun 30];10. Available from: /pmc/articles/PMC9372609/
132. Bains SS, Dubin JA, Green C, Herzenberg JE, McClure PK. Infection rates and risk factors with magnetic intramedullary lengthening nails. J Orthop [Internet]. 2024 Sep 1 [cited 2024 Jun 30];55:124–8. Available from: https://pubmed.ncbi.nlm.nih.gov/38706586/
133. Tschurl J, Shadi M, Kotwicki T. Hip Joint Stability during and after Femoral Lengthening in Congenital Femoral Deficiency. Children 2024, Vol 11, Page 500 [Internet]. 2024 Apr 22 [cited 2024 Jun 30];11(4):500. Available from: https://www.mdpi.com/2227-9067/11/4/500/htm
134. Song MH, Kim TJ, Chang AS, Song HR. Wire bending development and progression during Ilizarov system tibial lengthening in skeletally immature patients with achondroplasia. J Orthop Translat. 2020 Nov 1;25:73–9.
135. Sax O, Conway J, Standard S, Assayag M, Herzenberg J, McClure P. Risk factors for focal osteolysis in a stainless-steel limb-lengthening device. Journal of Limb Lengthening & Reconstruction. 2021;7(1):19.
136. Bayram S, Yıldırım AM, Eralp L, Şen C, Kocaoğlu M, Balci Hİ. The Relationship Between Limb Lengthening Rate and Callus Quality in Patients with Achondroplasia. Indian J Orthop [Internet]. 2022 Nov 1 [cited 2024 Jun 30];56(11):1891–6. Available from: https://pubmed.ncbi.nlm.nih.gov/36310565/
137. Sax OC, Hlukha LP, Kowalewski KA, Herzenberg JE, McClure PK. Does Focal Osteolysis in a PRECICE Stryde Intramedullary Lengthening Nail Resolve after Explantation? Children (Basel) [Internet]. 2022 Jun 1 [cited 2024 Jun 30];9(6). Available from: https://pubmed.ncbi.nlm.nih.gov/35740797/
138. Leblanc C, Rölfing JD, Langlais T, Sales de Gauzy J, Accadbled F. No osteolysis at the telescopic junction of 128 FITBONE lengthening nails. Orthop Traumatol Surg Res [Internet]. 2023 May 1 [cited 2024 Jun 30];109(3). Available from: https://pubmed.ncbi.nlm.nih.gov/36470369/
139. Reif TJ, Geffner A, Hoellwarth JS, Fragomen AT, Rozbruch SR. Precice Stryde® Magnetic Internal Lengthening Nail does not Impair Bone Healing Despite Radiographic and Clinical Symptoms. Strategies Trauma Limb Reconstr [Internet]. 2023 May 1 [cited 2024 Jun 30];18(2):94–9. Available from: https://pubmed.ncbi.nlm.nih.gov/37942435/

140. Iobst CA, Frost MW, Rölfing JD, Rahbek O, Bafor A, Duncan M, et al. Radiographs of 366 removed limb-lengthening nails reveal differences in bone abnormalities between different nail types. Bone Joint J [Internet]. 2021 Nov 1 [cited 2024 Jun 30];103-B(11):1731–5. Available from: https://pubmed.ncbi.nlm.nih.gov/34414785/
141. Liu Q, Liu Z, Guo H, Wang M, Liang J, Zhang Y. A comparative study of bone union and nonunion during distraction osteogenesis. BMC Musculoskelet Disord [Internet]. 2022 Dec 1 [cited 2024 Jun 30];23(1). Available from: https://pubmed.ncbi.nlm.nih.gov/36461051/
142. Gigi R, Hemo Y, Danino B, Ovadia D, Segev E. Changes in the femoral osteotomy level coefficient and neck shaft angle during limb lengthening with an intramedullary magnetic nail. Arch Orthop Trauma Surg [Internet]. 2022 Aug 1 [cited 2024 Jun 30];142(8):1739–42. Available from: https://pubmed.ncbi.nlm.nih.gov/33555401/
143. Liu Q, Mei H, Zhu G, Liu Z, Guo H, Wang M, et al. Early Pixel Value Ratios to Assess Bone Healing During Distraction Osteogenesis. Front Bioeng Biotechnol [Internet]. 2022 Jul 12 [cited 2024 Jun 30];10:1. Available from: /pmc/articles/PMC9315284/
144. Tomaszewski R, Wiktor Ł, Kler J, Pethe K, Gap A. Results of Femoral Elongation Treatment Using Electromagnetic Intramedullary Nail. Preliminary Report. Ortop Traumatol Rehabil [Internet]. 2020 Jun 30 [cited 2024 Jun 30];22(3):173–9. Available from: https://pubmed.ncbi.nlm.nih.gov/32732445/
145. Kitoh H, Mishima K, Matsushita M, Nishida Y, Ishiguro N. Early and late fracture following extensive limb lengthening in patients with achondroplasia and hypochondroplasia. Bone Joint J [Internet]. 2014 Sep 1 [cited 2024 Jun 30];96-B(9):1269–73. Available from: https://pubmed.ncbi.nlm.nih.gov/25183602/
146. Aranovich AM, Stogov M V., Tushina N V., Kireeva EA. Changes in the antioxidant/prooxidant balance in patients with acquired shortening of the lower leg bones at the stages of the Ilizarov lengthening procedure. Genij Ortopedii. 2022;28(3):386–91.
147. Balci Hİ, Anarat FB, Bayram S, Eralp L, Şen C, Kocaoğlu M. Does the technique of limb lengthening affect physeal growth in patient with achondroplasia? Comparison of the simultaneous and consecutive tibia and femur lengthening with external fixators. J Pediatr Orthop B [Internet]. 2023 Jan 1 [cited 2024 Jun 30];32(1):60–5. Available from: https://pubmed.ncbi.nlm.nih.gov/36125888/
148. Villalobos MA, Veneziano G, Iobst C, Miller R, Walch AG, Roth C, et al. Regional Anesthesia for Pain Management After Orthopedic Procedures for Treatment of Lower Extremity Length Discrepancy. J Pain Res [Internet]. 2020 [cited 2024 Jun 30];13:547–52. Available from: https://pubmed.ncbi.nlm.nih.gov/32214843/
149. Mittal A, Allahabadi S, Jayaram R, Nalluri A, Callahan M, Sabharwal S. What Factors Correlate With Length of Stay and Readmission After Limb Lengthening Procedures? A Large-database Study. Clin Orthop Relat Res [Internet]. 2022 Sep 1 [cited 2024 Jun 30];480(9):1754–63. Available from: https://pubmed.ncbi.nlm.nih.gov/35353078/
150. Flourish | Data Visualization & Storytelling [Internet]. [cited 2024 Jul 1]. Available from: https://app.flourish.studio/login
