# Supplementary material for: Outcomes and outcome measurement instruments in lower-limb lengthening surgery: a scoping review to inform core outcome set development
Source: Acta Orthop. 2024 Nov 29;95:715–22. doi: 10.2340/17453674.2024.42488 (PMC11605704; doi:10.2340/17453674.2024.42488)
Supplement: Supplementary file 5 [file ActaO-95-42488-s5.pdf]

Appendix 4. Outcome measurement instruments and complication classification systems

**Outcome Measurement Instruments (OMIs)**	**Number of studies**	**Physician-reported**	**Patient-reported**	**Citation**
(Tegner-) Lysholm Knee Scoring Scale	2		+	(1)
(Tegner) Activity Score	1		+	(2)
APLES Children	1		+	(3)
ASAMI grading system- functional results	9	+		(4)
ASAMI grading system-bone results	8	+		(4)
Catagni classification	1	+		(5)
Catagni’s evaluation form (for outcome)	1	+	+	(6)
Catagni’s evaluation form (for psychological outcome)	1		+	(6)
Chappell et al.	1	+		(7)
CHU-9D (Child health utility 9D)	1		+	(8)
Classification by Simpson and Kenwrigth of Fractures	3	+		(9)
Classification of Callus by Li et al.	5	+		(10)
Donnan classification	1	+		(11)
EQ-5D-3L questionnaire	1		+	(12)
EQ-5D-Y (EuroQol 5D youth)	1		+	(13)
EQ-5D-VAS	2	+	+	(14)
Goniometer	2	+		(15)
The World Health Organization (WHO) pain scale	1		+	(16)
LD-SRS	3		+	(17)
Limb symmetry index	1	+		(18)
Lower Extremity Functional Scale (LEFS)	1		+	(19)
Paley functional score	5	+	+	(20)
Patient Questionnaire for Paediatric and Adoslescent Limb Lengthening Surgical technique and treatment course	1		+	(21)
PROMIS Domain-Pain intensity	1		+	(22)
PROMIS-Global mental health	1		+	(22)
PROMIS-Global physical health	1		+	(22)

PROMIS-Pain Interference	1		+	(22)
PROMIS-Physical Function	1		+	(22)
RUST score	1	+		(23)
Fracture classification by Sax et al.	4	+		(24)
The body image quality of life inventory (BIQLI)	1		+	(25)
The International Cartilage Repair Society (ICRS) scoring system	1	+		(26)
The International Knee Documentation Committee (IKDC) Score	1	+		(27)
The Knee Society Score System	1	+	+	(28)
The modified Kitaoka score	1	+	+	(29)
The multidimensional body-self relations questionnaire	1		+	(30)
The Pediatric Quality of Life Inventory version 4.0 generic core scales (The PedsQL)	2		+	(31)
The situational inventory of body-image dysphoria (SIBID)	1		+	(32)
The Zung Self-Rating Anxiety Scale (SAS)	1		+	(33)
4 single-legged hop tests	1	+		(34)
30-second sit-to-stand test (30sSTS)	1	+		(35)
Visual Analog Scale (VAS)	7		+	(36)
Visual inspection	1	+		(37)
WHOQOL- BREF	1		+	(38)
Checketts-Otterburn Grading System	3	+		(39)
Saleh Scott Classification System	1	+		(40)
The AOFAS ankle–hindfoot scale	1	+	+	(41)
SF-12	1		+	(42)
SF-36	1		+	(43)
The International Physical Activity Questionnaire short form (IPAQ short)	1		+	(44)
SSI according to CDC-NHSN criteria	1	+		(45)
Gilette questionnaire	1		+	(46)
Functional evaluation system by Karlstrom-Olerurd	1	+	+	(47)
The Paley classification of recovery of limb function	1	+	+	(48)
The Paley classification of bone healing	1	+		(49)
The treatment result according to El-Gebaly et al.	1	+		(50)
The lengthening outcomes according to Trofimchuk et al.	1	+		(51)
The Cierny-Mader classification	1	+		(52)

Wong-baker faces pain rating scale	2		+	(53)
**Grand Total**	**102**	30	36	

**Complication classification systems**	**Number of studies**	**Citation**
Paley classification	28	(54)
The Lascombes Classification	9	(55)
The Black et al. classification of complications	5	(56)
Major complication-Minor complication	5	(57)
The modified Clavien-Dindo classification for orthopaedic surgery	3	(58)
Caton classification	2	(59)
Lee et al. classification of complications	1	(60)
Paley et al. with the modification of Schiedel et al.	1	(61)
Cherkashin et al. classification of complications	1	(62)
**Grand Total**	**55**	

References (Appendix 4):

1. Lysholm J, Gillquist J. Evaluation of knee ligament surgery results with special emphasis on use of a scoring scale. Am J Sports Med [Internet]. 1982 [cited 2024 Jun 17];10(3):150–4. Available from: https://pubmed.ncbi.nlm.nih.gov/6896798/
2. Tegner Y, Related JLCO and, 1985 undefined. Rating systems in the evaluation of knee ligament injuries. journals.lww.com [Internet]. [cited 2024 Jun 17]; Available from: https://journals.lww.com/corr/abstract/1985/09000/rating_systems_in_the_evaluation_of_knee_ligament.7.aspx
3. Bloemeke J, Sommer R, Witt S, Dabs M, Badia FJ, Bullinger M, et al. Piloting and psychometric properties of a patient-reported outcome instrument for young people with achondroplasia based on the International Classification of Functioning Disability and Health: the Achondroplasia Personal Life Experience Scale (APLES). Disabil Rehabil. 2019 Jul 17;41(15):1815–25.
4. Paley D, Catagni MA, Argnani F, Villa A, Battista Benedetti G, Cattaneo R. Ilizarov treatment of tibial nonunions with bone loss. Clin Orthop Relat Res [Internet]. 1989 [cited 2024 Jun 17];241(241):146–65. Available from: https://pubmed.ncbi.nlm.nih.gov/2924458/
5. Cardoso GS, Amorim R. Métodos de avaliação do regenerado ósseo*. Rev Bras Ortop (Sao Paulo). 2022 Sep 19;59(1):E1–9.
6. Catagni MA, Lovisetti L, Guerreschi F, Combi A, Ottaviani G. Cosmetic bilateral leg lengthening. Journal of Bone and Joint Surgery - Series B. 2005 Oct 1;87(10):1402–5.
7. Chappell TM, Ebert CC, McCann KM, Hutchinson BL, Rodriguez-Collazo E. Distal tibial distraction osteogenesis-An alternative approach to addressing limb length discrepancy with concurrent hindfoot and ankle reconstruction. J Orthop Surg Res [Internet]. 2019 Jul 30 [cited 2024 Jun 17];14(1):1–10. Available from: https://josr-online.biomedcentral.com/articles/10.1186/s13018-019-1264-0

8. Stevens K. Valuation of the child health utility 9D index. Pharmacoeconomics [Internet]. 2012 Dec 23 [cited 2024 Jun 17];30(8):729–47. Available from: https://link.springer.com/article/10.2165/11599120-000000000-00000
9. Simpson AHRW, Kenwright J. Fracture after distraction osteogenesis. J Bone Joint Surg Br [Internet]. 2000 [cited 2024 Jun 17];82(5):659–65. Available from: https://pubmed.ncbi.nlm.nih.gov/10963161/
10. Li R, Saleh M, Yang L, Coulton L. Radiographic classification of osteogenesis during bone distraction. Journal of Orthopaedic Research [Internet]. 2006 Mar 1;24(3):339–47. Available from: https://doi.org/10.1002/jor.20026
11. Donnan LT, Saleh M, Rigby AS, McAndrew A. Radiographic Assessment of Bone Formation in Tibia During Distraction Osteogenesis. Journal of Pediatric Orthopaedics [Internet]. 2002;22(5). Available from: https://journals.lww.com/pedorthopaedics/fulltext/2002/09000/radiographic_assessment_of_bone_formation_in_tibia.15.aspx
12. EuroQol--a new facility for the measurement of health-related quality of life. Health Policy [Internet]. 1990 [cited 2024 Jun 17];16(3):199–208. Available from: https://pubmed.ncbi.nlm.nih.gov/10109801/
13. Wille N, Badia X, Bonsel G, Burström K, Cavrini G, Devlin N, et al. Development of the EQ-5D-Y: a child-friendly version of the EQ-5D. Qual Life Res [Internet]. 2010 Aug [cited 2024 Jun 17];19(6):875–86. Available from: https://pubmed.ncbi.nlm.nih.gov/20405245/
14. EuroQol - a new facility for the measurement of health-related quality of life. Health Policy (New York). 1990 Dec 1;16(3):199–208.
15. Galal S, Shin J, Principe P, Khabyeh-Hasbani N, Mehta R, Hamilton A, et al. STRYDE versus PRECICE magnetic internal lengthening nail for femur lengthening. Arch Orthop Trauma Surg. 2021 May 13;142:3.
16. Ventafridda V, Saita L, Ripamonti CI, Conno F De. WHO guidelines for the use of analgesics in cancer pain. Int J Tissue React [Internet]. 1985;7 1:93–6. Available from: https://api.semanticscholar.org/CorpusID:20635621
17. Fabricant P, Borst E, Green S, Marx R, Fragomen A, Rozbruch Sr. Validation of a modified Scoliosis Research Society instrument for patients with limb deformity: The limb deformity-Scoliosis Research Society (LD-SRS) score. Journal of Limb Lengthening & Reconstruction [Internet]. 2016 [cited 2024 Jun 17];2(2):86. Available from: https://journals.lww.com/jllr/fulltext/2016/02020/validation_of_a_modified_scoliosis_research.6.aspx
18. Barber-Westin S, Noyes Frank MD, Mangine R, McCloskey, Hartman. Quantitative Assessment of Functional Limitations in Normal and Anterior Cruciate Ligament-Deficient Knees. Clin Orthop Relat Res. 1990 Jun 1;255:204–14.
19. Binkley JM, Stratford PW, Lott SA, Riddle DL. The Lower Extremity Functional Scale (LEFS): Scale Development, Measurement Properties, and Clinical Application. Phys Ther [Internet]. 1999 Apr 1 [cited 2024 Jun 17];79(4):371–83. Available from: https://dx.doi.org/10.1093/ptj/79.4.371
20. Paley D, Herzenberg JE, Paremain G, Bhave A. Femoral lengthening over an intramedullary nail. A matched-case comparison with Ilizarov femoral lengthening. JBJS. 1997;79(10):1464–80.
21. Iliadis AD, Palloni V, Wright J, Goodier D, Calder P. Pediatric Lower Limb Lengthening Using the PRECICE Nail: Our Experience With 50 Cases. J Pediatr Orthop [Internet]. 2021 Jan 1 [cited 2024 Jun 17];41(1):e44–9. Available from: https://pubmed.ncbi.nlm.nih.gov/32947442/
22. Heath MR, Shin TJ, Mehta R, Principe PS, MacKie AT, Fragomen A, et al. Patients With Lower Limb Deformity Report Worse Quality of Life Than Control Subjects Regardless of Degree of Deformity. JAAOS Global Research & Reviews [Internet]. 2021 Aug 10 [cited 2024 Jun 17];5(8). Available from: /pmc/articles/PMC8357251/
23. Leow JM, Clement ND, Tawonsawatruk T, Simpson CJ, Simpson AHRW. The radiographic union scale in tibial (RUST) fractures: Reliability of the outcome measure at an independent centre. Bone Joint Res [Internet]. 2016 Apr 1 [cited 2024 Jun 17];5(4):116. Available from: /pmc/articles/PMC5009237/
24. Sax O, Conway J, Standard S, Assayag M, Herzenberg J, McClure P. Risk factors for focal osteolysis in a stainless-steel limb-lengthening device. Journal of Limb Lengthening & Reconstruction. 2021;7(1):19.

25. Cash TF, Fleming EC. The impact of body image experiences: development of the body image quality of life inventory. Int J Eat Disord [Internet]. 2002 [cited 2024 Jun 17];31(4):455–60. Available from: https://pubmed.ncbi.nlm.nih.gov/11948650/
26. Brittberg M, Winalski CS. Evaluation of Cartilage Injuries and Repair. JBJS [Internet]. 2003;85(suppl_2). Available from: https://journals.lww.com/jbjsjournal/fulltext/2003/00002/evaluation_of_cartilage_injuries_and_repair.8.aspx
27. Higgins LD, Taylor MK, Park D, Ghodadra N, Marchant M, Pietrobon R, et al. Reliability and validity of the International Knee Documentation Committee (IKDC) Subjective Knee Form. Joint Bone Spine. 2007 Dec 1;74(6):594–9.
28. Insall JN, Dorr LD, Scott RD, Scott WN. Rationale of The Knee Society clinical rating system, 1993. Available from: http://www kneesociety org/web/pdfs/knee_society_rationale_article pdf.
29. Huang Q, Ma T, Ren C, Xu Y, Li M, Wang Q, et al. Shortening and re-lengthening versus bone transport for the treatment of distal tibial periarticular post-traumatic defects. Sci Rep [Internet]. 2022;12(1):16303. Available from: https://doi.org/10.1038/s41598-022-20760-0
30. Brown TA, Cash TF, Mikulka PJ. Attitudinal body-image assessment: factor analysis of the Body-Self Relations Questionnaire. J Pers Assess [Internet]. 1990 [cited 2024 Jun 17];55(1–2):135–44. Available from: https://pubmed.ncbi.nlm.nih.gov/2231236/
31. Varni JW, Seid M, Kurtin PS. PedsQL™ 4.0: Reliability and Validity of the Pediatric Quality of Life Inventory™ Version 4.0 Generic Core Scales in Healthy and Patient Populations. Med Care [Internet]. 2001;39(8). Available from: https://journals.lww.com/lww-medicalcare/fulltext/2001/08000/pedsql__4_0__reliability_and_validity_of_the.6.aspx
32. Cash TF. The situational inventory of body-image dysphoria: psychometric evidence and development of a short form. Int J Eat Disord [Internet]. 2002 Nov [cited 2024 Jun 17];32(3):362–6. Available from: https://pubmed.ncbi.nlm.nih.gov/12210651/
33. Zung WWK. A Rating Instrument For Anxiety Disorders. Psychosomatics. 1971 Nov 1;12(6):371–9.
34. Noyes FR, Barber SD, Mangine RE. Abnormal lower limb symmetry determined by function hop tests after anterior cruciate ligament rupture. Am J Sports Med [Internet]. 1991 [cited 2024 Jun 17];19(5):513–8. Available from: https://pubmed.ncbi.nlm.nih.gov/1962720/
35. Jones CJ, Rikli RE, Beam WC. A 30-s chair-stand test as a measure of lower body strength in community-residing older adults. Res Q Exerc Sport [Internet]. 1999 Jun 1 [cited 2024 Jun 17];70(2):113–9. Available from: https://pubmed.ncbi.nlm.nih.gov/10380242/
36. Delgado DA, Lambert BS, Boutris N, McCulloch PC, Robbins AB, Moreno MR, et al. Validation of Digital Visual Analog Scale Pain Scoring With a Traditional Paper-based Visual Analog Scale in Adults. J Am Acad Orthop Surg Glob Res Rev [Internet]. 2018 Mar 1 [cited 2024 Jun 17];2(3). Available from: /pmc/articles/PMC6132313/
37. Femino JD, Nelson SC, Agulnik M, Zuckerman LM. All-Internal Limb Lengthening with Simultaneous Corrective Osteotomy After Internal Hemipelvectomy: A Case Report. JBJS Case Connect [Internet]. 2022 May 12 [cited 2024 Jun 17];12(2). Available from: https://pubmed.ncbi.nlm.nih.gov/36099533/
38. Harper A, Power M, Orley J, Herrman H, Schofield H, Murphy B, et al. Development of the World Health Organization WHOQOL-BREF quality of life assessment. The WHOQOL Group. Psychol Med [Internet]. 1998 May [cited 2024 Jun 17];28(3):551–8. Available from: https://pubmed.ncbi.nlm.nih.gov/9626712/
39. Checketts RG, MacEachem AG, Otterbum M. Pin Track Infection and the Principles of Pin Site Care. In: De Bastiani G, Apley AG, Goldberg A, editors. Orthofix External Fixation in Trauma and Orthopaedics [Internet]. London: Springer London; 2000. p. 97–103. Available from: https://doi.org/10.1007/978-1-4471-0691-3_11
40. Saleh M, Scott BW. Pitfalls and complications in leg lengthening: the Sheffield experience. In: Seminars in Orthopaedics. WB SAUNDERS COMPANY; 1992. p. 207.

41. Kitaoka HB, Alexander IJ, Adelaar RS, Nunley JA, Myerson MS, Sanders M. Clinical rating systems for the ankle-hindfoot, midfoot, hallux, and lesser toes. Foot Ankle Int [Internet]. 1994 [cited 2024 Jun 17];15(7):349–53. Available from: https://pubmed.ncbi.nlm.nih.gov/7951968/
42. WARE JE, KOSINSKI M, KELLER SD. A 12-Item Short-Form Health Survey: Construction of Scales and Preliminary Tests of Reliability and Validity. Med Care [Internet]. 1996;34(3). Available from: https://journals.lww.com/lww-medicalcare/fulltext/1996/03000/a_12_item_short_form_health_survey__construction.3.aspx
43. Ware J, Snoww K, MA K, BG G. SF36 Health Survey: Manual and Interpretation Guide. Lincoln, RI: Quality Metric, Inc, 1993. 1993 Jan 1;30.
44. Craig CL, Marshall AL, Sjöström M, Bauman AE, Booth ML, Ainsworth BE, et al. International physical activity questionnaire: 12-country reliability and validity. Med Sci Sports Exerc [Internet]. 2003 Aug 1 [cited 2024 Jun 17];35(8):1381–95. Available from: https://pubmed.ncbi.nlm.nih.gov/12900694/
45. Bains SS, Dubin JA, Green C, Herzenberg JE, McClure PK. Infection rates and risk factors with magnetic intramedullary lengthening nails. J Orthop [Internet]. 2024 Sep 1 [cited 2024 Jun 17];55:124–8. Available from: https://pubmed.ncbi.nlm.nih.gov/38706586/
46. Novacheck TF, Stout JL, Tervo R. Reliability and Validity of the Gillette Functional Assessment Questionnaire as an Outcome Measure in Children with Walking Disabilities. Journal of Pediatric Orthopaedics [Internet]. 2000;20(1). Available from: https://journals.lww.com/pedorthopaedics/fulltext/2000/01000/reliability_and_validity_of_the_gillette.17.aspx
47. Karlström G, Olerud S. Fractures of the Tibial Shaft A Critical Evaluation of Treatment Alternatives. Clin Orthop Relat Res [Internet]. 1974;105. Available from: https://journals.lww.com/clinorthop/fulltext/1974/11000/fractures_of_the_tibial_shaft_a_critical.6.aspx
48. Xie J, Zhao G, Yasheng T, Chen H, Amuti N, Maimaitirexiati M, et al. Ilizarov bone transport to treat infected nonunion of long bones: a multicenter retrospective cohort study. J Int Med Res [Internet]. 2021 [cited 2024 Jun 18];49(3):1–8. Available from: /pmc/articles/PMC8166389/
49. Paley D, Maar DC. Ilizarov bone transport treatment for tibial defects. J Orthop Trauma. 2000;14(2):76–85.
50. Ghaly HM, El-Rosasy MAM, Marei AE, El-Gebaly OA. Simultaneous femoral and tibial lengthening for severe limb length discrepancy in fibular hemimelia. J Orthop Surg Res [Internet]. 2023;18(1):844. Available from: https://doi.org/10.1186/s13018-023-04229-y
51. Trofimchuk V, Dossanov B, Lozovoy V, Khmyzov S, Dossanova A, Angelov A, et al. Quality of Life in Children With Achondroplasia Undergoing Paired Limb Lengthening With an External Fixator and Modified Distraction Control: Observational Nonrandomized Study. JMIR Rehabil Assist Technol. 2024 Jan 24;11:e49261.
52. Mader JT, Ortiz M, Calhoun JH. Update on the diagnosis and management of osteomyelitis. Clin Podiatr Med Surg. 1996;13(4):701–24.
53. Wong DL, Baker CM. Wong-Baker faces pain rating scale. Pain Management Nursing. 2012;
54. Paley D. Problems, obstacles, and complications of limb lengthening by the Ilizarov technique. Clin Orthop Relat Res. 1990;250(1):81–104.
55. Lascombes P, Popkov D, Huber H, Haumont T, Journeau P. Classification of complications after progressive long bone lengthening: proposal for a new classification. Orthopaedics & Traumatology: Surgery & Research. 2012;98(6):629–37.
56. Black SR, Kwon MS, Cherkashin AM, Samchukov ML, Birch JG, Jo CH. Lengthening in congenital femoral deficiency a comparison of circular external fixation and a motorized intramedullary nail. Journal of Bone and Joint Surgery - American Volume. 2014 Sep 2;97(17):1432–40.
57. Frommer A, Roedl R, Gosheger G, Niemann M, Turkowski D, Toporowski G, et al. What Are the Potential Benefits and Risks of Using Magnetically Driven Antegrade Intramedullary Lengthening Nails for Femoral Lengthening to Treat Leg Length Discrepancy? Clin Orthop Relat Res [Internet]. 2022 Apr 1 [cited 2024 Jun 18];480(4):790–803. Available from: https://pubmed.ncbi.nlm.nih.gov/34780384/
58. Sink EL, Leunig M, Zaltz I, Gilbert JC, Clohisy J, Beaulé P, et al. Reliability of a complication classification system for orthopaedic surgery. journals.lww.com [Internet]. 2012 [cited 2024 Jun 18];470(8):2220–6. Available from: https://journals.lww.com/clinorthop/FullText/2012/08000/Reliability_of_a_Complication_Classification.22.aspx

59. Caton J, Dumont P, … JBR de chirurgie, 1985 undefined. Intermediate results of a series of 33 cases of leg lengthening using H. Wagner's technic. pubmed.ncbi.nlm.nih.gov [Internet]. [cited 2024 Jun 18]; Available from: https://pubmed.ncbi.nlm.nih.gov/4081158/
60. Lee DH, Kim S, Lee JW, Park H, Kim TY, Kim HW. A Comparison of the Device-Related Complications of Intramedullary Lengthening Nails Using a New Classification System. Wiley Online LibraryDH Lee, S Kim, JW Lee, H Park, TY Kim, HW KimBioMed Research International, 2017•Wiley Online Library [Internet]. 2017 [cited 2024 Jun 18];2017. Available from: https://onlinelibrary.wiley.com/doi/abs/10.1155/2017/8032510
61. Schiedel FM, Vogt B, Tretow HL, Schuhknecht B, Gosheger G, Horter MJ, et al. How precise is the PRECICE compared to the ISKD in intramedullary limb lengthening? Reliability and safety in 26 procedures. Taylor & FrancisFM Schiedel, B Vogt, HL Tretow, B Schuhknecht, G Gosheger, MJ Horter, R RödlActa orthopaedica, 2014•Taylor & Francis [Internet]. 2014 [cited 2024 Jun 18];85(3):293–8. Available from: https://www.tandfonline.com/doi/abs/10.3109/17453674.2014.913955
62. Cherkashin A, Samchukov M, … JBJ of P, 2015 undefined. Evaluation of complications of treatment of severe Blount's disease by circular external fixation using a novel classification scheme. journals.lww.com [Internet]. [cited 2024 Jun 18]; Available from: https://journals.lww.com/jpo-b/FullText/2015/03000/Evaluation_of_complications_of_treatment_of_severe.9.aspx
